# Antibiotics create a shift from mutualism to competition in human gut communities with a longer-lasting impact on fungi than bacteria

**DOI:** 10.1186/s40168-020-00899-6

**Published:** 2020-09-12

**Authors:** Bastian Seelbinder, Jiarui Chen, Sascha Brunke, Ruben Vazquez-Uribe, Rakesh Santhaman, Anne-Christin Meyer, Felipe Senne de Oliveira Lino, Ka-Fai Chan, Daniel Loos, Lejla Imamovic, Chi-Ching Tsang, Rex Pui-kin Lam, Siddharth Sridhar, Kang Kang, Bernhard Hube, Patrick Chiu-yat Woo, Morten Otto Alexander Sommer, Gianni Panagiotou

**Affiliations:** 1 Leibniz Institute for Natural Product Research and Infection Biology—Systems Biology and Bioinformatics, Hans Knöll Institute, Adolf-Reichwein-Straße 23, 07745 Jena, Germany; 2 Department of Medicine, State Key Laboratory of Pharmaceutical Biotechnology, The University of Hong Kong, Hong Kong, SAR China; 3 Leibniz Institute for Natural Product Research and Infection Biology—Microbial Pathogenicity Mechanisms, Hans Knöll Institute, Adolf-Reichwein-Straße 23, 07745 Jena, Germany; 4 Novo Nordisk Foundation Center for Biosustainability, Technical University of Denmark, Kemitorvet 220, DK-2800 Lyngby, Denmark; 5 Department of Microbiology, Li Ka Shing Faculty of Medicine, The University of Hong Kong, Pokfulam, Hong Kong; 6 Emergency Medicine Unit, Li Ka Shing Faculty of Medicine, The University of Hong Kong, Pokfulam, Hong Kong; 7 State Key Laboratory of Emerging Infectious Diseases, The University of Hong Kong, Pokfulam, Hong Kong; 8 Collaborative Innovation Center for Diagnosis and Treatment of Infectious Diseases, The University of Hong Kong, Pokfulam, Hong Kong

## Abstract

**Background:**

Antibiotic treatment has a well-established detrimental effect on the gut bacterial composition, but effects on the fungal community are less clear. Bacteria in the lumen of the gastrointestinal tract may limit fungal colonization and invasion. Antibiotic drugs targeting bacteria are therefore seen as an important risk factor for fungal infections and induced allergies. However, antibiotic effects on gut bacterial-fungal interactions, including disruption and resilience of fungal community compositions, were not investigated in humans. We analysed stool samples collected from 14 healthy human participants over 3 months following a 6-day antibiotic administration. We integrated data from shotgun metagenomics, metatranscriptomics, metabolomics, and fungal ITS2 sequencing.

**Results:**

While the bacterial community recovered mostly over 3 months post treatment, the fungal community was shifted from mutualism at baseline to competition. Half of the bacterial-fungal interactions present before drug intervention had disappeared 3 months later. During treatment, fungal abundances were associated with the expression of bacterial genes with functions for cell growth and repair. By extending the metagenomic species approach, we revealed bacterial strains inhibiting the opportunistic fungal pathogen *Candida albicans*. We demonstrated in vitro how *C*. *albicans* pathogenicity and host cell damage might be controlled naturally in the human gut by bacterial metabolites such as propionate or 5-dodecenoate.

**Conclusions:**

We demonstrated that antibacterial drugs have long-term influence on the human gut mycobiome. While bacterial communities recovered mostly 30-days post antibacterial treatment, the fungal community was shifted from mutualism towards competition.

Video abstract.

## Background

The human gut microbiome is a complex ecosystem of bacteria, fungi, archaea, and phages [1]. The majority of research has focused on the bacterial part of the gut microbiome and their role in health and disease [2–4]. However, the critical role of fungi in host homeostasis remains is less well studied. Fungal dysbiosis may increase symptoms of inflammation, especially in the gut lumen [5]. Treating mice with fluconazole, an antifungal drug, increases the immune response and severity of experimentally induced colitis [6] but also induced allergic airway disease [7]. Fluconazole seems to substantially impact only certain types of fungi such as *Candida*, but not *Aspergillus* species [6].

Antibiotic treatment has a well-established detrimental effect on the composition of gut bacteria [8–11], but the effect on the fungal community is less clear. Nevertheless, antibiotic use is linked to overgrowth of particular fungi at multiple body sites [7, 8, 12]. Noverr et al. used a murine model to induce development of airway allergies by enriching for *Candida* and *Aspergillus* species in the gut followed by antibiotic treatment [10]. Theoretically, commensal bacteria may limit fungal colonization by production of antifungal compounds [13], competition for available nutrients, cellular contact, chemotaxis, or physiochemical changes to the local environment [14, 15]. Fungi defend themselves by secreting molecules, forming biofilms or forming mutualistic bonds with other bacteria. *Candida albicans*, for example, secretes the metabolite farnesol which interferes with the quorum-sensing of *Pseudomonas aeruginosa* [14, 15]. However, *C*. *albicans* can also enhance biofilm formation by *Staphylococcus aureus* in vitro. *Pseudomonas fluorescens* promotes the growth of the mycorrhizal fungus *Laccaria bicolor* in soil. Which bacterial-fungal interactions take place in the gastrointestinal tract of humans remains to be investigated. To date, the complex community of gut microbes is thought to be coevolved to maintain relative homeostasis in healthy humans [16].

Defining gut fungal consortia and their stability, resilience, and dynamics may reveal cause-effect relationships with bacteria. Although evidence is available on bacterial-fungal interactions in the gut at the taxonomic level [13–15], we do not have a comprehensive understanding of how bacterial functions influence the growth of particular fungi. Bacterial microbiome studies were often performed by amplifying the DNA of the ribosomal 16S gene. However, metagenome shotgun sequencing allows species- and sometimes even strain-level taxonomic classification, as well as the estimation of gene functions [17–19]. Furthermore, gene expression in microbial communities is not strictly matched with metagenomic potential [20]. Often, studies neglect the high transcriptional activity of some less-abundant species to metabolic functions.

In order to better understand the microbiome, we provide data to follow both, the bacterial and fungal communities of the lower human gastrointestinal tract over 3 months after antibiotic treatment concomitantly. We provide an overview of how the mycobiome and its interactions with the bacterial microbiome change and we reveal dependencies of specific fungal species from bacterial functions at DNA and RNA levels.

## Results

### Antibiotic treatment triggers long-lasting dynamics at fungal species level

**Fig. 1:**
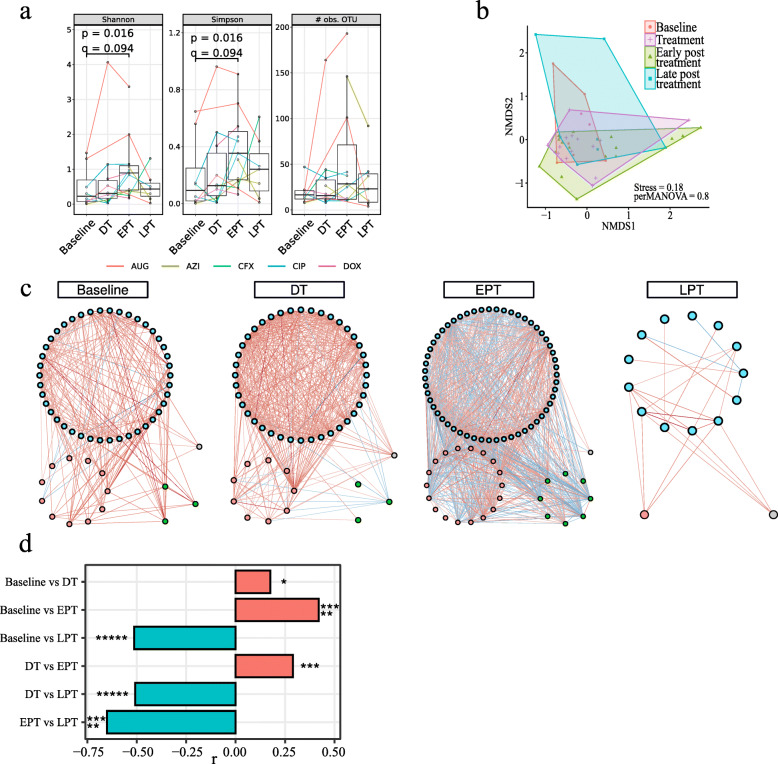
Antibiotic treatment induces fungal competition. Statistical testing by Wilcoxon signed-rank tests with *p* values adjusted for multiple testing using false discovery rate (FDR) (*q* = FDR[p]). Not significant, ns: *q* ≥ 0.05; *q < 0.05; **q < 0.01; ***q < 1e− 3; ****q < 1e− 4; *****q < 1e− 5. **a**, **b** Diversity analysis of samples from treated participants using PIPITS operational taxonomic units (OTU) relative abundances. **a** Boxplots showing Shannon (left) and Gini-Simpson indexes (middle) and species richness (right) with median (centerlines), first and third quartiles (box limits), and 1.5× interquartile range (whiskers). No significant changes were observed (*q* < 0.05). **b** Non-metric dimensional scaling of Bray-Curtis distance as a measure of beta diversity. No significant differences (*p* < 0.05) were found between time points using PERMANOVA. **c**, **d** Co-abundance network analysis using BAnOCC. Only OTUs with prevalence 10% and significant correlations (95% credibility interval) with |*r*| ≥ 0.3 were used for network construction. Networks were created independently for baseline, during (DT), early post (EPT), and late post treatment (LPT) to show temporal changes in fungal communities. **c** Fungal networks. Node colour indicates fungal phyla. Blue, Ascomycota; red, Basidiomycota; green, Mucoromycotina; grey, unknown. Edge colour indicates correlation type. Red, positive; blue, negative. **d** Network properties. Bar plots show number of nodes that increased and decreased in node degree centrality

We included 14 healthy human participants, 12 receiving the antibiotic intervention and 2 controls. Stool samples were collected at 4–6 time points per participants. We used 5 different antibiotics (one for each pair of treated participants). Samples were collected 15 days before administration of antibiotics (baseline), at 4 and 6 days of treatment (during treatment [DT]), 15 and 30 days after (early post treatment [EPT]), and 90 days after treatment (late post treatment [LPT]). We built high-quality libraries for ITS2 sequencing for 59 of 74 available stool samples. We estimated the fungal relative abundance using the PIPITS pipeline [21]. ITS sequences were clustered into operational taxonomic units (OTU) and taxonomically annotated using Mothur [22]. Antibiotic treatment led to a significant increase in species-level fungal alpha diversity during early post treatment compared to baseline (Fig. 1a; two-sided Wilcoxon rank-sum test, *p* = 0.016, *q* = 0.094). Controls showed a considerable increase as well, although statistical significance could not be estimated due to the number of subjects (*n* = 2). At the level of individual antibiotic drugs (Suppl. Fig. 1), Augmentin and ciprofloxacin more than doubled baseline diversity. In contrast, changes for doxycycline and azithromycin were mild. Beta diversity using Bray-Curtis was not significantly different between time points in treated samples (Fig. 1b; PERMANOVA, *p* > 0.05).

We subsequently investigated differences in fungal genera relative abundance over time. *Candida* genus increased 15-fold from baseline to treatment (*q* = 0.004; Suppl. Table 1). *Candida* increase was observed for all antibiotics except Augmentin (Suppl. Fig. 2). At the species level, results were more distinct (Suppl. Table 2) and for this analysis, we considered only prevalent fungal species (defined as present in 15% of samples). Comparing relative abundance changes from baseline to during treatment, only *Saccharomyces sydowii* decreased significantly (*q* < 0.05). However, the opportunistic pathogen *Candida albicans* tended to increase 7-fold (*q* < 0.07) and was affected the most by Augmentin and doxycycline (Suppl. Fig. 3). Furthermore, *C*. *albicans* was detected in nine participants after treatment even though in only five at baseline.

Twenty-three species changed significantly from treatment to early post treatment periods (*q* < 0.05), many of which were not present before or after treatment. Many common fungi like *Saccharomyces* spp., opportunistic pathogens such as *C*. *albicans*, *C*. *parapsilosis*, and *Malassezia restricta*—a fungus recently connected to pancreatic cancer [23]—decreased in abundance, whereas less common fungi such as *Candida boidinii* increased in abundance. A minor decrease in abundance of *C*. *albicans* was also observed in controls, but not nearly as much.

To test for long-lasting changes, we compared relative abundances at baseline to late post treatment and found six species with significant changes. We further noticed that only 14 fungal species passed the prevalence filter at baseline and late post treatment, whereas up to 44 were observed during and early post treatment, suggesting that antibiotics temporarily created a niche for less common fungal species. In summary, the number of detected, prevalent species more than doubled during treatment and early post treatment, but these species had not successfully colonized 3 months later. Most changes were found within the first month after treatment, implying a delayed response of the fungal community to the treatment. Over one third of the fungal species present before treatment showed significant changes even 90 days after treatment.

#### Antibiotic treatment increases co-exclusion in fungal communities

We evaluated changes induced in the mycobiome from antibiotic administration by creating co-abundance networks based on ITS abundances. Networks were created for baseline and for during, early, and late post treatment periods (Fig. 1d). Only significant edges (95% credibility) with absolute correlation of at least 0.3 were retained. Generally, we found significant correlations within and between *Ascomycota*, *Basidiomycota*, and *Mucoromycotina* species. At baseline, we found mostly positive correlations (240 positive and 10 negative) among 57 fungal species. During treatment, the number of correlations almost doubled (406 positive, 17 negative), whereas at early post treatment, correlation numbers doubled again. In contrast to the previous networks, more than half of the significant correlations were negative (399 negative, 550 positive), implying a major switch from mutualistic relationships at baseline and during treatment to competition between fungal species as they try to re-establish a stable community. We also observed these negative correlation patterns within and between fungal phyla. At late post treatment, this conflict persisted. Most co-abundance patterns had disappeared—only 25 correlations among 15 fungal species remained. We confirmed these trends by testing for significant changes in node degree centrality (Fig. 1e; Suppl. Table 3).

In conclusion, based on diversity, abundance, and network analysis, we observed that gut fungal communities started to change alongside the bacterial communities during treatment. Many fungi failed to colonize successful and competition emerged during early post treatment, leading to changes that lasted 90 days after treatment. The human mycobiome became more stochastic, leading to strikingly less co-abundance patterns among fungal species. These findings indicated that the gut mycobiome was not resilient enough to recover from the influence of antibiotics within 3 months.

### Changes in functional metagenomic diversity from antibiotics are not strictly followed by changes in metatranscriptomic diversity

We characterized the subjects’ microbiomes at baseline (Suppl. Fig. 4) and found that bacterial communities were dominated by bacteria from the *Bacteroidetes* and *Firmicutes* phyla but with strong variation in ratio, as expected in healthy individuals [16, 24]. In line with previous studies, we observed a significant decrease in bacterial species alpha diversity (Suppl. Fig. 5). Ciprofloxacin had the strongest (− 40%) and cefuroxime the weakest negative effect (− 5%), whereas controls only an insignificant increase (2%) (Suppl. Fig. 6). Beta diversity was significantly different during antibiotic treatment, but not in controls. In addition, we found that antibiotic treatment had the strongest influence on moderately abundant bacterial species (Suppl. Fig 5). We then estimated bacterial growth using GRiD [25] (Suppl. Table 4). In antibiotic-treated subjects, median growth of species decreased significantly during treatment compared to baseline as expected (*p* = 0.009, *r* = − 0.56, Suppl. Fig. 7). Interestingly, the number of species with growth rate greater 1 increased significantly (*p* = 0.0016, *r* = 0.68).

**Fig. 2:**
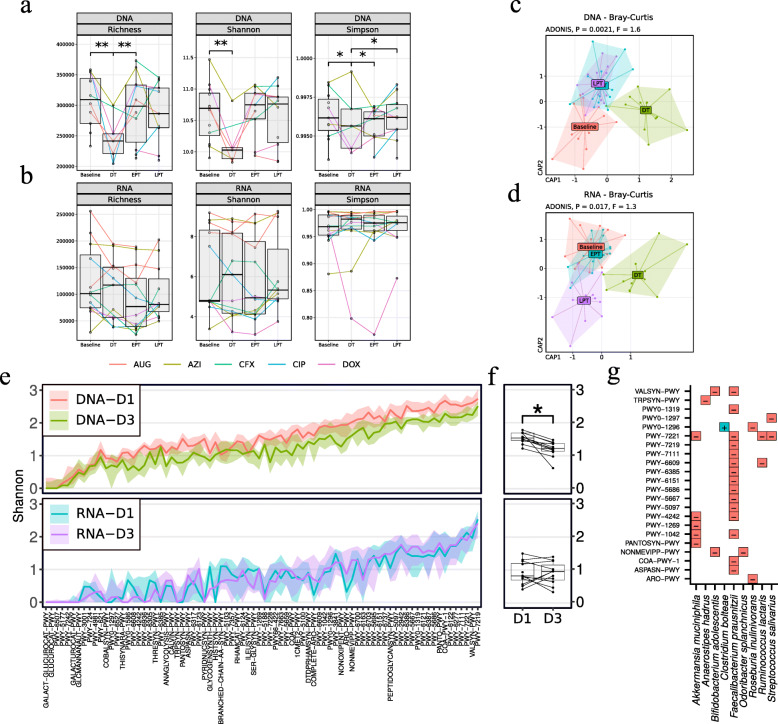
Metagenomic contributional alpha diversity of metabolic function is severely reduced by antibiotic treatment. Diversity analysis of metagenomic and metatranscriptomic samples from participants using HUMAnN2 relative abundances. **a**, **b** Alpha diversity of gene family relative abundances using **a** metagenomic and **b** metatranscriptomic data. Boxplots show species richness (left), Shannon (middle), and Gini-Simpson indexes (right) at 15 days before treatment (baseline), during (DT), and 30 days (EPT) and 90 days post treatment (LPT). Median (centerlines), first and third quartiles (box limits), and 1.5× interquartile range (whiskers) are shown. Lines between boxes connect same-donor samples. Statistical testing was by Wilcoxon signed-rank test with *p* values adjusted for multiple testing using false discovery rate (*q*; *0.01 ≤ *p* < 0.05). **c**, **d** Constrained ordination of Bray-Curtis dissimilarity based on gene family abundances measured using principle coordinate analysis (PCoA). We used distance-based redundancy analysis to show the explained variance by sample time points while accounting for participant-specific influence. **e**–**g** Contributional Shannon diversity of MetaCyc pathways of baseline and treatment samples. **e** Top, metagenomic and bottom, metatranscriptomic contributions. Mean (solid lines) and first and third quartiles (transparent ribbons) are shown. **f** Mean contributional diversity per participant per time point for DNA (top) and RNA (bottom). **g** Species with significantly increased (blue, +) and decreased (red, −) contribution to pathways based on two-sided Wilcoxon signed-rank test adjusted for compositionality (*q* < 0.1)

We subsequently investigated functional changes based on bacterial gene family abundance in the metatranscriptome and the metagenome. Alpha diversity of relative DNA gene family abundance was significantly reduced during treatment compared to baseline (Fig. 2a; *q* < 0.05), but not in controls (Suppl. Fig. 8). Despite the changes at the DNA level, the alpha diversity for relative RNA gene family abundance did not change significantly between time points (Fig. 2b; *q* > 0.05).

We investigated differences in beta diversity of gene family abundances based on Bray-Curtis dissimilarity (Fig. 2c, d). We performed ordination and statistical testing using distance-based redundancy analysis (dbRDA) using “subject id” as a constrained variable and “sample time” as an independent variable. In treated subjects, both DNA and RNA functional abundances showed significant differences in centroids between timepoints (Fig. 2c, d; DNA: *F* = 2, *p* = 0.002, Df = 5; RNA: *F* = 1.6, *p* = 0.037, Df = 5). This was not observed in controls (Suppl. Fig. 9). Pairwise dbRDA revealed a significant difference from baseline to treatment in DNA functional abundance (*F* = 3.13, *q* = 0.014, Df = 1; Suppl. Table 5). For RNA abundance, we observed only a trend (*F* = 1.6, *p* = 0.085, Df = 1). Overall, these findings implied that the genetic potential of the bacterial community was reduced during treatment as expected. However, gene expression changes were considerably less compared to the metagenome and not as consistent among participants. Similarly, antibiotic treatment had no significant effect on the transcriptional activity of the core and variable metabolic pathways (as defined in [20]; Suppl. Fig. 10). In agreement with previous findings [20], the metatranscriptome was much more dynamic than the metagenome.

#### Diversity of bacterial contribution to metabolic pathways is systematically reduced by antibiotics

We investigated if the contribution of species to a given pathway changed significantly over time [26] (Fig. 2g, h; Suppl. Fig. 11). By DNA, the median contributional alpha diversity of antibiotic-treated participants decreased significantly from baseline to treatment (Shannon: log_2_ fold-change [lf2] = − 0.4; *q* = 0.015; Simpson: lf2 = − 0.24; *q* = 0.015). Controls showed no significant changes (*q* > 0.05). In contrast, we did not observe significant changes in alpha diversity measures for RNA (*q* > 0.05). We further investigated if the contribution of single bacterial species to metabolic pathways changed significantly between time points. We implemented a compositionality test as described in Palleja et al. [27], considering all pathways, and found 9 bacterial species whose contribution significantly changed (*q* < 0.1; Fig. 2i). Important gut commensal bacteria including *Akkermansia muciniphila*, *Faecalibacterium prausnitzii*, *Odoribacter splanchnicus*, and *Bifidobacterium adolescentis* contributed less during treatment. A decline of such butyrate-producing species following antibiotic treatment has been observed before [11, 27]. In contrast, the multiantibiotic-resistant bacterium *Clostridium bolteae* [28] contributed more.

### Antibiotic treatment lastingly reduced bacterial-fungi interactions

We increased the functional resolution of bacterial species using the metagenomic species (MGS) concept [29], which allows identification of taxonomically unidentified bacterial species. We further improved the method to identify some bacteria at the strain level based on their genetic potential. In contrast to previous studies, we used HUMAnN2 [19] gene family profiles as references in accordance with a published protocol [30]. HUMAnN2-derived profiles allowed us to retrieve MGS with high purity (i.e. more than 95% of genes in an MGS group originated from the same species; Suppl. Table 6). We then identified 26 MGS with significant change in relative abundance during treatment compared to baseline (Suppl. Fig. 12; Suppl. Table 7), which was not observed in controls. Six of these had species-level annotation and were consistently decreased independent of the antibiotic drug used (*Ruminococcus lactaris*, *Dialister invisus*, *Odoribacter splanchnicus*, *Bacteroidetes bacterium ph8*, *Akkermansia muciniphila*, *Bifidobacterium adolescentis*; full list in Suppl. Figs. 13 and 14).

**Fig. 3:**
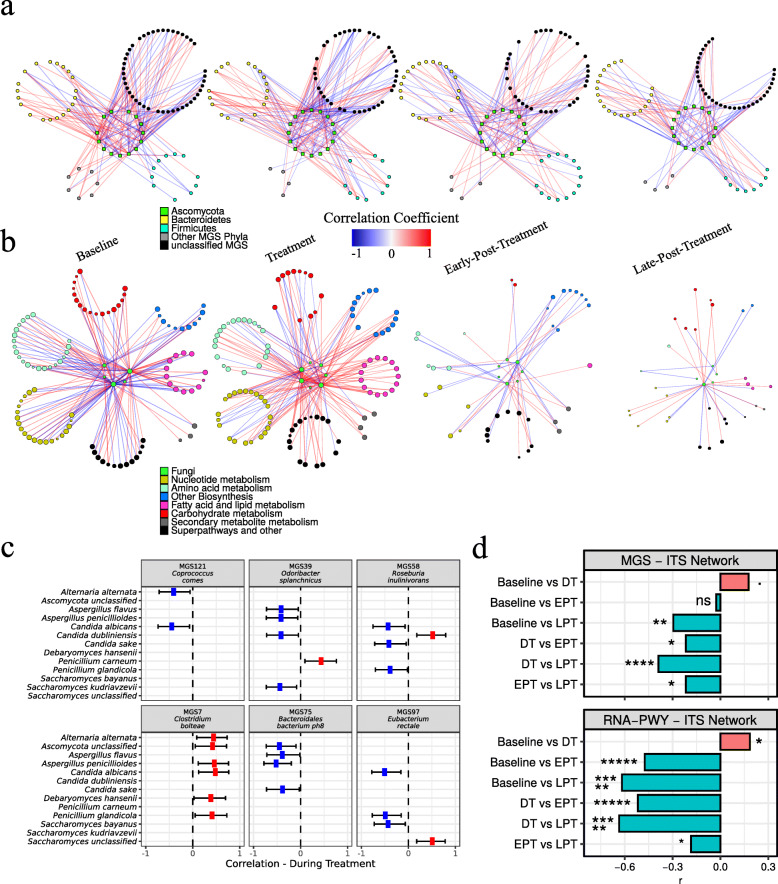
Cross-kingdom interactions among fungi, bacteria species, and pathway expression. **a**, **b** Co-abundance networks at indicated time points using BAnOCC with **a** 25% and **b** 50% prevalence filter. Only significant edges are shown (based on 95% credibility interval) with |*r*| ≥ 0.3. Negative correlations (blue), positive correlations (red). Networks are left (baseline) to right (late post treatment). **a** Correlations among fungal and bacterial species based on metagenomic species (MGS) and internal transcribed spacer (ITS) relative abundance. **b** Correlations among fungal species and pathway expression based on HUMAnN2 RNA pathway and internal transcribed sequence relative abundance. Superpathways and other pathways that did not fit into the six major categories were grouped as “other”. **c** Estimated correlation between bacterial and fungal species during treatment. Positive (red), negative (blue). Error lines show 95% confidence intervals. **d** Effect size of node degree change. *r* values change from − 1 (100% decrease) to 1 (100% increase). (Top) MGS and ITS relative abundances. (Bottom) RNA-PWY and ITS relative abundances. Statistical testing for significant changes in node degree was performed using a two-sided Wilcox signed-rank test. *P* values were adjusted for multiple testing using FDR. Node degree was determined independently for baseline, during (DT), early post (EPT), and late post treatment (LPT). Significance is indicated by symbols (ns, *q* ≥ 0.05; *q < 0.05; **q < 0.01; ***q < 1e− 3; ****q < 1e− 4; *****q < 1e− 5)

We combined MGS and ITS relative abundance data and used BAnOCC [31] to infer intra- and cross-kingdom associations among bacterial and fungal species. We created co-abundance networks at the species level for baseline and for during, early post and late post treatment periods independently as described above (Suppl. Fig. 15). In order to find significant changes in the structure of co-abundance networks, we compared differences in node degree. The degree of a node is defined by the number of significant correlations with that node. Hence, an increase in node degree implies an increase of potential interactions, i.e. an increase of potentially relevant effects. To study changes in bacterial-fungal interactions, we tested for significant differences in node degree centrality considering only cross-kingdom correlation (Fig. 3a, c; Suppl. Table 8). We observed a temporal increase in node degree during treatment compared to baseline (*q* = 0.055). From during to early post treatment, this degree dropped (*q* = 0.0185) and decreased further at late post treatment (*q* = 0.0185). To find lasting changes, we compared baseline against late post treatment and found significantly reduced degree (*q* = 0.00134). Considering these results in addition to the loss of correlations observed in the fungal network, we conclude that antibiotic treatment was a triggering event for disturbances in bacterial-fungal interactions. These disturbances ultimately drove gut bacteria and fungi towards independence.

We looked more closely at co-abundance patterns involving bacterial species with significant changes in abundance or pathway contribution during treatment (Fig. 3c; all significant correlation in Suppl. Table 9). *C*. *bolteae* increased in relative abundance during treatment and correlated positively with many fungal species during treatment, such as the opportunistic pathogen *C*. *albicans*, or the mycotoxin producers *Aspergillus penicillioides* and *Penicillium glandicola*. In contrast, *O*. *splanchnicus* was persistently negatively correlated with opportunistic pathogens from the genera *Candida*, *Aspergillus*, and *Alterna*. *O*. *splanchnicus* is part of the healthy gut community but rarely investigated in terms of its role. *Roseburia inulinivorans* was negatively associated to opportunistic pathogens *C*. *albicans*, *C*. *sake*, and *P*. *glandicola*. Low *Roseburia* abundance was associated with higher glucose levels and ulcerative colitis [32, 33]. Likewise, *Eubacterium rectale* was negatively associated with *C*. *albicans* and *P*. *glandicola*. Depending on the diet, *Eubacterium rectale* decreased glucose and insulin levels [34]. Notably, butyrate-producing species were negatively associated with at least one opportunistic fungal pathogen.

At last, we considered bacterial-fungal correlations together with MGS abundance changes during treatment and fungal abundance changes in early post treatment. Bacterial species with decreased relative abundance and negative correlation to a fungus that showed an increased abundance were considered competitors. For example, *O*. *splanchnicus* was decreased during treatment, and showed negative correlation to *C*. *albicans*. A list of possible bacterial-fungal competitors is shown in Suppl. Table 10.

#### Prevalent fungi correlated with pathway expression during treatment

We investigated relationships among metabolic pathway expression levels (MetaCyc database—PWY; metatranscriptomic abundance) and fungal ITS abundance (Fig. 3b) by creating co-abundance networks analogous to the bacteria-fungi network. We tested for significant changes in node degree considering only correlations between fungal OTUs and pathway expression. From baseline to treatment, node degree increased (*q* = 0.0185; Fig. 3d). Most correlations were positive (146 of 189) during treatment in contrast to baseline (82 of 153). Hence, and despite the increase of variance of metatranscriptome diversity during treatment, we still observed co-abundance with fungal species during treatment. This observation suggested a mutual influence between the fungal community and expression of bacterial metabolic pathways. About one third of correlations at baseline involved *C*. *albicans* and one third involved *Saccharomyces*. After treatment, node degree dropped significantly to below baseline levels (during vs. early post: *q* = 4e− 11; early vs. late post: *q* = 0.0185; baseline vs. late post: *q* = 3e− 15). Almost all *C*. *albicans* co-abundance patterns were lost at 90 days post treatment, with *Saccharomyces* genus accounting for over 70% of remaining correlations (24 of 31). Overall, *Saccharomyces* appears to be more resilient with respect to bacterial metabolic pathways expression than other prevalent fungi.

We then increased our resolution by focusing on correlations between fungal OTU abundance and pathways in broader functional categories (Suppl. Fig. 16; Suppl. Table 11). We observed a significant increase in node degree from baseline to treatment for pathway functions in nucleotide metabolism (*q* = 0.026) and biosynthetic pathways (e.g. for vitamins, tetrapyrroles, NAD) (*q* = 0.043). Almost all correlations were positive. We found no significant changes in remaining categories (*q* < 0.1; metabolism of amino acids, carbohydrates, fatty acids and lipids, secondary metabolites). Node degree dropped significantly after treatment in all categories except secondary metabolite metabolism (*q* < 0.05).

Since our treatment targeted bacteria, we expected the bacterial community to respond to the selective pressure with strong, directed changes in pathway expression. Most metatranscriptomic changes appeared to be stochastic. Yet we still observed mostly positive co-abundance patterns between fungal abundance and bacterial pathway expression during treatment, especially with functions required for bacterial growth. Even though correlations do not imply causations, when performed on multiple different levels, they can still offer significant insights. The results suggested a common origin for changes in the mycobiome and pathway expression: if gut fungi generally take advantage of reduced complexity in the bacterial community, we would expect an increase in fungal diversity. However, we observed no systematic change. Highly abundant and adapted fungi may still overgrow. In both scenarios, we would expect an increase in negative correlations between fungal and bacterial abundances during treatment but found mostly positive correlations. Generally, antibiotics drove the mycobiome alongside the microbiome, leading to a temporal increase in fungal richness, but also increased fungal competition subsequently. On the long run, antibiotic treatment broke down most of the inferred relationships between bacteria and fungi, as shown by diverging mycobiomes 3 months after treatment.

### Key bacterial species and molecular mediators of *Candida albicans* colonization

Our ITS data showed at least one *C*. *albicans* read per participant over 112 days but with varying relative abundance from 0 to 42%. *C*. *albicans* was detected during treatment even if it was not detected at baseline, as in other studies [12, 35], confirming antibiotic treatment as risk factor for colonization and overgrowth of this fungus. Furthermore, *C*. *albicans* significantly decreased 2 weeks after treatment, implying the indirect impact on its growth by the microbiome. We searched for metabolites detected in the human gut that may inhibit or promote *C*. *albicans* growth. We performed metabolomics analyses on a subset of 15 stool samples and calculated Spearman’s correlations for the relative abundance of each metabolite and relative abundance of *C*. *albicans* by ITS (Suppl. Fig. 17). Based on these findings, we performed *C*. *albicans* growth assays in defined medium containing serial dilutions of selected metabolites. With several metabolites, including 4-hydroxybenzoic acid and 8,11,14-eicosatrienoic acid, we observed only minor growth reductions at the highest concentrations (Suppl. Fig. 18). More pronounced growth reduction occurred with adipic acid, aminoadipic acid, and ornithine, but fungi still grew with high concentrations of these metabolites. In contrast, propionic acid, acetic acid, and cis-5-dodecenoic acid fully inhibited growth at a range of concentrations. We then tested if the substances directly damaged human cells. Using a human vaginal cell line (A431) without *C*. *albicans*, the bile acid lithocholate (LCA) and cis-5-dodecenoic acid showed limited cytotoxicity (Suppl. Fig. 19). No other substances caused detectable cell damage.

**Fig. 4:**
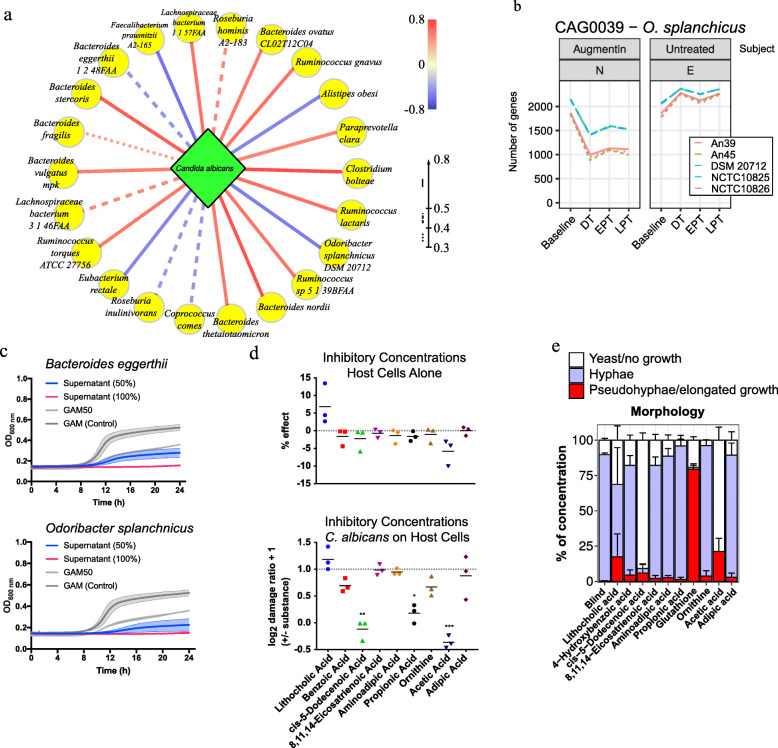
*Candida albicans* growth promotors and inhibitors. **a** Bacterial species co-abundant with *C*. *albicans*. Line colours and type indicate correlation coefficients. **b** Example using *Odoribacter splanchnicus* for genomic strain inference from metagenomic species (MGS) reads. Strains were inferred for each time point (*x*-axis) from number of genes with 0.5 reads per base (*y*-axis) per-reference genome. Data are from the antibiotic-treated participant N and untreated subject E. The number of genes coverage for each tested *O*. *splanchnicus* strains are shown. DSM 20712 was identical with another strain labelled NCTC10825. **c** Growth rate inhibition of *C*. *albicans* strain SC5314 cultivated with 50% and 100% bacterial supernatant (from *Bacteroides eggerthii* and *Odoribacter splanchnicus*) compared to control of medium (mGAM) only. **d** Damage of human vaginal epithelial cells (A431) based on release of lactate dehydrogenase (LDH) with metabolites at inhibitory concentrations. Grey lines, zero effect. Positive values imply cell damage. (Top) Human cells cultured without *C*. *albicans*. Effect compared to untreated cells. (Bottom) Cells co-cultured with *C*. *albicans*. Values are relative to damage caused by *C*. *albicans* without additional metabolites. Negative values imply less cell damage. **e** Composition of morphology of *C*. *albicans* cultures quantified by concentration of morphological types. Some metabolites cause atypical formation of hyphae-like structures (“Pseudohyphae/elongated yeast chains”). Some inhibited the formation of filaments or disrupted growth in general (“Yeast/no growth”)

Next, we assayed the effect on human cells by *C*. *albicans* in presence of the same metabolites. At higher concentrations, when in vitro fungal growth was reduced, human cell damage decreased with the short-chain fatty acids (SCFAs) propionic (*p* < 0.05) and acetic acid (*p* < 0.001). Acetic or cis-5-docenoic acid (*p* < 0.01) almost fully abolished cell damage by *C*. *albicans*. Benzoic acid reduced damage to a lesser extent (*p* = 0.051). Since the morphology (yeast or hyphal cells) is critical for its damaging potential, we investigated if the substances also led to morphological changes in *C*. *albicans* (Fig. 4e). On high concentrations, hyphae formation and growth were almost completely suppressed by 5-dodecenoic and acetic acid. 5-dodecenoate also reduced hyphae formation under growth-permitting concentration. Glutathione only allowed formation of chains elongated yeasts resembling pseudohyphae. LCA partially suppressed hyphal growth at the high concentration, resulting in high numbers of pseudohyphae and yeast cells.

These metabolites that affect *C*. *albicans* growth negatively may also promote the growth of its fungal competitors, such as *Saccharomyces* spp., *Penicillium* spp. and *Aspergillus* spp. Therefore, we repeated the correlation analysis with the corresponding OTUs (Suppl. Table 12). For each fungal species, we found several metabolites with positive correlation. Considering metabolites negatively affecting *C*. *albicans*, only 2-methyl butanoic acid and 3-hydroxy butyric acid were found to be significantly positively correlated with *Penicillium spinulosum* and LCA with *Aspergillus flavus*. Still, promotive effects on other fungal species need to be verified in future work.

#### Bacterial supernatant inhibits *C*. *albicans* growth

We investigated which gut bacteria might be the main direct or indirect producers or contributors of these compounds in our human participants. We correlated metabolite concentrations with MGS relative abundances (Suppl. Fig. 20) and focused on positive associations. We looked at species that correlated with multiple, different metabolites. *Bacteroides coprophilus* correlated with aminoadipic acid and acetate; *C*. *comes* with 4-hydroxybenzoic acid, 5-dodecenoate, and glutathione; *F*. *prausnitzii* with 4-hydroxybenzoic acid; *E*. *lenta* with 5-dodecenoate and eicosatrienoic acid; *B*. *eggerthii* with 5-dodecenoate and eicosatrienoic acid; and *O*. *splanchnicus* with acetate. All six species correlated with LCA or its derivates.

Our correlation methods helped us to pinpoint bacteria that may promote or inhibit *C*. *albicans* growth (Fig. 4a). For testing these predicted associations in vitro, we selected bacterial strains based on sufficient confidence in our strain-level inference in addition to significant correlation to *C*. *albicans*. We performed the strain identification directly from the MGS profiling. Instead of strain detection methods using single-nucleotide polymorphisms (e.g. StrainPhlAn [18], metaSNV [36], ConStrains [37]), we adopted a strategy based on gene content as in PanPhlAn [38]. We therefore analysed reads corresponding to a specific MGS. For example, gene coverage for *O*. *splanchnicus* strains for two participants (N, E; Fig. 4b; Suppl. Fig. 21) showed that both subjects had the highest coverage for strain DSM 20712, so we selected DSM 20712 for in vitro assays. In the end, we were interested in bacterial strains for which we found significant correlation with inhibitory metabolites, significant correlation with *C*. *albicans*, and high confidence from the strain inference. Based on these results, we selected *Bacteroides eggerthii* and *Odoribacter splanchnicus* for further in vitro experiments.

We determined the antifungal effect of metabolites produced by selected bacterial strains using their sterilized culture supernatants as growth medium for *C*. *albicans*. We measured *C*. *albicans* growth using 100% or 50% supernatant diluted in modified Gifu anaerobic media (mGAM) (Fig. 4c). Percentage inhibition was compared to optimal growth conditions in fresh medium. *C*. *albicans* growth was significantly inhibited by supernatants from *B*. *eggerthii* (50% growth) or *O*. *splanchnicus* (40%). Using 100% bacterial supernatants had stronger effects, showing that inhibition was proportional to supernatant dilution (Suppl. Fig. 22). We tested two additional *C*. *albicans* strains to exclude that observed effects were strain specific but saw no differences (Suppl. Fig. 22). *B*. *eggerthii* and *O*. *splanchnicus* also inhibited *C*. *albicans* growth in pairwise in vitro co-culturing experiments (Suppl. Fig. 23).

Finally, we analysed the supernatant of these species to characterize their metabolic capabilities that may relate to *C*. *albicans* growth (Suppl. Fig. 24-25). We included the supernatant from *Ruminococcus* [Blautia] *torques* as positive control, since this species was shown to have positive effect on *C*. *albicans* growth previously (Mirhakkak et al., 2020, under review) and correlated positively with *C*. *albicans* in our study. Compared to quality control samples, *O*. *splanchnicus* supernatant contained high concentrations of butyric acid (7-fold relative conc.), which suppresses *C*. *albicans* growth in vitro [39]. But we also measured elevated levels of the growth suppressing metabolites adipic and aminoadipic acid, and ornithine. In contrast, *B*. *eggerthii* supernatant contained elevated levels of acetic acid (1.6-fold), formic acid (3-fold), and hexanoic acid (2-fold). The full growth-inhibiting metabolites 2-methyl-propanoic acid and propanoic acid were also found in supernatants of *O*. *splanchnicus* and *B*. *eggerthii*, but roughly 3-times higher in *B*. *eggerthii*. In contrast, *R*. *torques* produced only formic acid in higher abundance (1.25-fold), but almost none of the strong inhibitory SCFA. Altogether, the supernatant analysis shows that propionate, ornithine, and benzoic acid are effective inhibitors of *C*. *albicans* growth, and these compounds were likely produced by *B*. *eggerthii* and *O*. *splanchnicus* also in the human gut.

## Discussion

Mouse models can offer some advantages for studying competitive relationships between gut bacteria and fungi. Previous studies have shown that antibiotics induce fungal overgrowth in the murine gut lumen [12, 35, 40]. However, antibiotic doses used in mice experiments create an almost germ-free environment after treatment, which is unlikely to apply to the human gut with clinical use of antibiotics. Furthermore, the mice gut microbiome and human gut microbiome differ considerably [41–43]. For example, many *Firmicutes* spp., which represent major colonizers of the human gut, cannot efficiently colonize the murine gut. Sovran et al. showed that *Enterobacteriaceae* play an important role for bacteria-fungi interactions in the murine gut [44]. In their study, *Enterobacteriaceae* accounted for 40 to 65% of reads in Vancomycin treated mice. We investigated the relative abundance of *Enterobacteriaceae* spp. in our human subjects. However, the accumulated relative abundance of *Enterobacteriaceae* spp. for most samples was below 1% before, during and after treatment (median 0.02%; except for Augmentin with 13%), making it difficult to assess whether *Enterobacteriaceae* were relevant for bacterial-fungal interactions in the human gut (Suppl. Fig. 26).

In this study, we investigated if fungal overgrowth was induced in the human gastrointestinal tract under physiological conditions. We present evidence that changes on fungal abundance at the species level are highly dynamic in the lower human gastrointestinal tract. Even though gut bacteria and fungi successfully prevented several temporarily detected fungi from colonization the lumen lastingly, we found significant alternations to the relative abundance of several fungi even 90 days after antibiotic treatment.

We used 5 different broad-spectrum antibiotics which are commonly used to treat human diseases [45]. Recent work by Maier et al. [46] addresses the issue that most knowledge of antibiotic drugs and their bacterial targets is based on pathogens and not the commensal microbiome. In a large screening of 144 different antibiotics and the 40 most common gut microbial strains, most antibiotics inhibited growth of all tested bacterial strains. Only *Clostridium* showed consistent resistance to many drugs. Indeed, some bacterial species are stronger or less affected depending on the antibiotic used. We investigated how much these expected differences apply to our data. Effect sizes varied, but overall, most of the significant changes were independent of specific antibiotic drugs. Because of our small cohort size, we cannot assess if the differences in effect sizes are due to differences in baseline communities or differential inhibition of the drugs. More work is required by using bigger cohorts as well as other antibiotic drugs with narrower targets.

Co-abundance networks inferred mutual relationships between fungal species at baseline and during treatment. Post treatment, however, competition emerged. Furthermore, we observed far fewer co-abundance patterns between fungi and bacteria in early and late post treatment periods, indicating profound decline in bacterial-fungal interactions. Overall, we found the fungal community to be less resilient than the bacterial. Based on these data, we hypothesize that the dominant gut fungi of healthy individuals were in balance with gut bacteria. Antibiotic administration induced profound changes to gut bacteria that translated into changes in fungal abundance that lasted until the end of our study period. Indeed, these results must be considered with caution, as we did not perform quantitative estimations of bacterial and fungal abundances. In most cases, relative abundance estimation does not allow inference of true direction of change [47]. For quantification, bacterial cells are counted by flow cytometry in addition to DNA sequencing or qPCR [47, 48]. However, broad-spectrum antibiotics decrease bacterial cell counts by 3 orders of magnitude [47]. We estimated bacterial growth in situ to show that bacterial growth was significantly impaired at the community level. Hence, significant decrease in relative abundance of species will likely be reflected in true abundance as well. In future work, increasing the number of study subjects will help to increase certainty in and resolution of the findings.

One of the largest knowledge gaps about the basic biology of gut microbial balance is the lack of comprehensive functional analyses. Metatranscriptome studies have found both more [20] and less [49] uniformity in individual participants’ profiles compared to respective metagenomes. Despite minor changes in beta diversity, we found no significant changes induced by antibiotic treatment in gene family alpha diversity, species contribution, or transcriptional activity of metabolic pathways. This result was most likely due to high variability in the metatranscriptome, as observed previously in healthy humans [20]. However, fungal abundance and bacterial growth may have influenced one another because mutual relations between fungal abundances and expression of bacterial functions for growth were inferred, especially during treatment. Because these patterns were not as pronounced before and after treatment, we identified antibiotic administration as the main driver of this change.

Understanding and finding microbial mediators of fungal pathogens may help to improve antifungal treatments. We focused our study on *C*. *albican*s, testing in vitro if growth was affected by compounds produced by two bacterial species, *B*. *eggerthii* and *O*. *splanchnicus*. Although the supernatant of each bacterium was used in combination with optimal *C*. *albicans* growth medium, the supernatants inhibited *C*. *albicans* growth considerably. Such a condition is plausible for the lower human intestine, because we expect most easily metabolizable compounds, e.g. carbohydrates, to be absorbed by the small intestine. Furthermore, the two species may be physically separated in the gut lumen. Some of the metabolites with clear growth reduction to *C*. *albicans* were found in bacterial supernatants. However, we cannot exclude potential promoting effects of other bacteria that could occur in the same vicinity.

A decline in gut bile acids and SCFA is linked to disease states [50, 51], but cause-effect mechanisms are less understood. We identified five metabolites that naturally occur in the human gut to effectively inhibit growth and/or lower hyphae formation, a key attribute of *C*. *albicans* virulence [52]. Acetate is a prototypical SCFA that dampens the immune response at higher concentrations [53]. The SCFA propionate plays an important role in immune regulation [54]. Lithocholate is a secondary bile acid and such secondary bile acids may inhibit *C*. *albicans* growth [50]. Glutathione is an antioxidant that dampens cell damage [55]. Cis-5-dodecenoic acid suppressed hyphae formation entirely. A similar compound, cis-2-dodecenoic acid, is produced by *Burkholderia cenocepacia* and strongly interferes with *C*. *albicans* growth [56, 57]. In contrast to previous studies [39, 51] we also show that acetate, 5-dodecenoic acid, and propionate also significantly reduced *C*. *albicans*-mediated host cell damage in vitro. These compounds could also support the growth of fungal *C*. *albicans* competitors. However, a correlation analysis between these metabolites and multiple different *Saccharomyces*, *Penicillium*, and *Aspergillus* spp. did not indicate that. Nevertheless, this needs to be experimentally verified in future work.

Several limitations should be highlighted. Observing gut bacterial and fungi concomitantly is difficult as long as bacterial and fungal abundances are estimated using two independent sequencing technologies. Improvements in correlation methods mitigate some of the resulting problems. Still, our correlation results regarding inter-kingdom species-species correlation could be improved in the future. Estimating cell counts per kingdom would further help to improve correlation estimates. Our findings are further limited to just the 5 antibiotic drugs used. Even though many significant findings seemed consistent across the drugs, increasing the number of patients for each drug would help to get more differentiated results. When studying mechanistic effects with respect to *C*. *albicans* growth, we could not simulate the complexity of the gut community. We aimed to find metabolic regulators, but the growth of fungi and bacteria in the gut is certainly based on a combination of several metabolic factors and environmental conditions. We looked at a variety of aspects from host cell damage to morphology, but these were still in vitro findings.

Our results indicated that antibiotic treatment has a longer-lasting impact on gut fungi than bacteria, driving fungal communities from mutualism to competition. This work also advanced MGS methods for resolving microbiome compositions and interactions. Of potential clinical relevance, we demonstrate how particular SCFAs and bile acids produced by gut bacteria restricted human cell damage from *C*. *albicans* but also show other compounds with considerable effects.

## Conclusions

Theoretically, bacteria and fungi compete for resources available on the gut lumen, but they may also support one another. In this study, we investigated the temporal, concomitant changes of gut bacteria and fungi in humans. We demonstrate that antibacterial drugs have long-term influence on the human gut mycobiome, driving fungal communities from mutualism to competition. We further show how metabolites produced by bacteria such as cis-5-dodecenoic acid may actively suppress pathogenicity of opportunistic fungi such as *C*. *albicans*. We thereby show that gut bacterial-fungal interactions are an important consideration for antibacterial treatment.

## Methods

### Study design

#### Human participants

Stool samples were gathered from 14 healthy adults, aged 18–65 years, from Denmark and Hong Kong. Samples were collected over 3–4 months. The Danish study was approved by the local ethics committee in Region Zealand, Denmark (SJ-383), and the Hong Kong study was approved by the Institutional Review Board of The University of Hong Kong/Hospital Authority Hong Kong West Cluster (UW 17-042). All work was performed in accordance with the Good Clinical Practice principles and the Helsinki Declaration. Written informed consent was obtained from all participants. Patient characteristics are described in (Suppl. Table 13). Subjects with any of the exclusion criteria below were not eligible for entry into the present study: (i) history of taking antibiotics over the last 6 months, (ii) receiving systemic antifungals/antifungal mouthwashes or probiotics concurrently, (iii) patients suffering from immunosuppressive conditions or taking immunosuppressants, and (iv) severe medical comorbidities requiring frequent hospitalization. Another cohort of six healthy, untreated individuals from Canada was acquired from a previous study from Raymond et al. [58].

#### Treatment

Of the participants, 12 were treated for 6 days with 1 antibiotic drug out of 5: doxycycline (tetracycline class), azithromycin (macrolide class), Augmentin (β-lactam class), ciprofloxacin (quinolone class), and cefuroxime (β-lactam class). Two untreated participants were used as controls.

#### Sampling

From each participant in the clinical study in Denmark, 6 stool samples were obtained: one 15 days before treatment (± 1 day), two during treatment (days 3 and 5 of treatment ± 1 day), and three at 15, 30, and 90 days after treatment (± 1 day). From each participant in the clinical study in Hong Kong, four stool samples were obtained at 7 days before treatment (± 1 day), day 6 of treatment, and 30 and 90 days after treatment. Collected samples were aliquoted and stored at − 80° immediately after collection until DNA extraction. Stool samples of control patients treated with placebo [58] were acquired before, 7 days, and 90 days after treatment.

### Metagenomics and metatranscriptomics sequencing

For participants in the clinical study in Denmark, bacterial metagenomics and metatranscriptomics raw data were obtained from Kang et al. (*in preparation*). Briefly, DNA was extracted using a MO BIO PowerMax Soil DNA Extraction Kit (MO BIO Laboratories, Inc) and purified with PowerClean Pro DNA Clean-Up Kits (MO BIO Laboratories, Inc.) according to the manufacturer’s protocol. For RNA, rRNA was depleted using a Ribo-Zero Gold rRNA removal kit—Epidemiology (Illumina). The remaining total RNA was extracted using a MO BIO PowerMicrobiome™ RNA Isolation Kit (MO BIO Laboratories, Inc.). RNA and DNA sequencing were performed on an Illumina HiSeq 2000 (PE125). For participants in the clinical study in Hong Kong, bacterial DNA and RNA were extracted from 200 mg aliquots of frozen stool by Beijing Genome Institute (BGI). DNA was extracted using an E.Z.N.A.® Stool DNA Kit according to the manufacturer’s protocol. For RNA, rRNA was depleted using a Ribo-Zero™ Magnetic Kit. The remaining total RNA was extracted using a RiboPure-Yeast Kit. All samples were sequenced on an Illumina HiSeq 4000 platform (Illumina, San Diego, California, USA; paired-end, insert size 350 bp, read length 150 bp for DNA and 100 bp for RNA).

### Internal transcribed spacer sequencing

All stool samples from both cohorts were processed by Novogene for internal transcribed spacer (ITS) sequencing. DNA was extracted using the following protocol: Stool samples were thoroughly mixed with 900 μL of CTAB lysis buffer. All samples were incubated at 65 °C for 60 min before being centrifuged at 12000×*g* for 5 min at 4 °C. Supernatants were transferred to fresh 2-mL microcentrifuge tubes and 900 μL of phenol:chloroform:isoamyl alcohol (25:24:1, pH = 6.7; Sigma-Aldrich) was added for each extraction. Samples were mixed thoroughly prior to being incubated at room temperature for 10 min. Phase separation occurred by centrifugation at 12,000×*g* for 15 min at 4 °C, and the upper aqueous phase was re-extracted with a further 900 μL of phenol:chloroform:isoamyl alcohol. Next, samples were centrifuged at 12,000×*g* for 10 min at 4 °C, and the upper aqueous phases were transferred to fresh 2-mL microcentrifuge tubes. The final extraction was performed with 900 μL of chloroform:isoamyl alcohol (24:1), and layer separation occurred by centrifugation at 12,000×*g* for 15 min at 4 °C. Precipitation of DNA was achieved by adding the upper phase from the last extraction step to 450 μL of isopropanol (Sigma-Aldrich) containing 50 μL of 7.5 M ammonium acetate (Fisher). Samples were incubated at − 20 °C overnight, although shorter incubations (1 h) produced lower DNA yields. Samples were centrifuged at 7500×*g* for 10 min at 4 °C, and supernatants were discarded. Finally, DNA pellets were washed three times in 1 mL of 70% (v/v) ethanol (Fisher). The final pellet was air-dried and re-suspended in 200 μL of 75 mM TE buffer (pH = 8.0; Sigma-Aldrich). The resulting fungal sequences were amplified using ITS2-F: 5′ GCATCGATGAAGAACGCAGC-3′ and ITS2-R: 5′ TCCTCCGCTTATTGATATGC-3′ primers [59, 60]. ITS2 amplicons were generated in three steps by PCR with 38 cycles: 98 °C 10s, 59 °C 10s, and 72 °C 30s followed by sequencing on the Illumina HiSeq platform (2 × 250 bp, Novogen, China).

### Metabolomics

For 4 participants, bile acid profiles and MicrobioMET profiles were assessed by Metabo-Profile (Shanghai, China) using aliquots of frozen stool. For bile acid profiles, bile acid-free matrix (BAFM) was obtained using the charcoal-stripping protocol. Calibrators and quality controls were prepared for the BAFM and processed as for extraction of bile acids from stool samples. About 10 mg prechilled zirconium oxide beads were added to 10 mg stool with 15 μl ultrapure water. To each sample, a 200-μl aliquot of prechilled acetonitrile/methanol containing 10 internal standards was added for homogenization. After centrifugation at 13,500 rpm and 4 °C for 20 min, 50 μl supernatant was transferred to 96-well plates. Acetonitrile/water (150 μl) was added for gentle shaking for 5 min before injection into an ultra-performance liquid chromatography column coupled to tandem mass spectrometry (UPLC-MS/MS) system to quantitate bile acids.

MicrobioMET profiles including aromatic phenols and indoles, phenolic acids, short-chain fatty acids and branched-chain amino acids, amino acids, and organic acids were quantitated using gas chromatography coupled to time-of-flight mass spectrometer (GC-TOFMS). Stool aliquots (50 mg) were homogenized with 300 μl NaOH (1 M) solution using a homogenizer and centrifuged at 13,500 rpm and 4 °C for 20 min. Supernatants (200 μl) were transferred into autosampler vials and residue extracted with 200 μl cold methanol. After a second homogenization and centrifugation, 167 μl supernatant was combined with the first supernatant in the autosampler vial. Extracts were capped and used for automated sample derivatization by a robotic multipurpose sample MPS2 with dual heads (Gerstel, Muehlheim, Germany). Samples pre-treated with sodium sulfate were shaken at 1500 rpm and 4 °C for 20 min and transferred to capped empty autosampler vials for the GC-TOFMS.

### Data processing

#### Quality control of sequence data

Quality control of raw reads (DNA, RNA) used a previously described pipeline [61]. Adapter sequences, low-quality bases (*Q* < 20), duplicated reads, reads shorter than 75 bp and reads mapping to the human genome with 95% coverage were filtered out. Computational scripts are at https://github.com/TingtZHENG/VirMiner/.

#### In situ bacterial growth rate estimation

Quality controlled FASTQ samples were sub-sampled to 2 million reads per sample. GRiD version 1.3 [25] was used with the corresponding stool database on sub-sampled samples to assess the growth bacterial strains. Default parameters were used but with minimum coverage threshold of 0.5 in order to investigate growth rates for different thresholds. After investigating the results, and as suggested by the GRiD authors, we continued with the growth estimates for strains with coverage 1.0 or higher. Statistical testing of (a) median growth rates and (b) the number of growing species was performed with a Wilcoxon signed-rank test. Normalized effect size *r* was estimated using the R package “rcompanion” and its function “wilcoxonPairedR”.

#### Abundance profiling

HUMAnN2 [19] version 0.11.1 was used to estimate gene family abundances in metagenomic DNA and RNA samples. Resulting reads per kilo-base (RPK) for gene family abundances at species level (including unclassified taxa) were further normalized by counts per million (CPM), resulting in a transcripts per kilo-base million (TPKM) like normalization.

PIPITS pipeline [21] version 1.4.5 was used for ITS with default parameters including quality filtering, read-pair merging, ITS2 filtering, and chimaera removal. Remaining reads were binned based on 97% similarity as operational taxonomic unit and aligned to the UNITE fungi database using Mothur classifier [22]. For further downstream analysis, all samples were normalized by cumulative sum scaling using MetagenomeSeq [62].

For bile acid profiles, raw data from UPLC-MS/MS were processed using QuanMET software (v1.0, Metabo-Profile) for peak integration, calibration and quantitation for each bile acid. The analyte concentration of unknown bile acid was calculated using a calibration curve.

For MicrobioMET profiles, raw data from the GC-TOFMS were processed using proprietary software XploreMET (v2.0, Metabo-Profile) for automatic baseline denosing, smoothing, peak picking, and peak signal alignment. MS-based quantitative metabolomics determined the concentration of unknown metabolites by comparing the unknown to a calibration curve. Abundance of MirobioMET profiles was calculated to minimize large individual variations in metabolites.

#### Metagenomic sequences from HUMAnN2 profiles

TPKM-normalized gene family abundances from DNA were clustered using mgs-canopy version 1.0 software (https://anaconda.org/bioconda/mgs-canopy). We used standard parameters except for a Pearson correlation coefficient cut-off of 0.95 instead of the default 0.9. Gene family clusters with at least 700 genes were considered metagenomic sequences (MGS). Taxonomic annotation of MGS used species annotation information available for each gene family. We calculated contributions of each species to an MGS (including unclassified taxa). An MGS was annotated to species level using the largest gene family distribution if the gene contribution of that species was at least 51% and the second largest species (a) was “unclassified” or (b) contributed at most 10%. MGS with more than 90% gene contribution from the same species were considered “pure” or “unambiguous”. Using a more stringent species assignment than the original method [29], from a total of 213 MGS, we obtained 80 with species-level assignment (Suppl. Table 6).

#### Genomic strains from MGS

MGS with species assignments were processed independently. Reads that (1) contributed to the abundance of an MGS, and (2) mapped to the inferred species (based on ChocoPhlAn reference [19]) were extracted. We used PanPhlAn [38] version 1.2.1.3 to create species-specific pangenomes based on reference genomes from the National Center for Biotechnology Information (Suppl. Table 14), mapped reads against the species pangenome, and calculated per-gene per-reference profiles. Gene abundance was normalized to reads per base. A gene was covered sufficiently if it had at least 0.5 reads per base. We accepted a strain reference if: (1) at least 90% of genes in the MGS were found to have sufficient coverage, and (2) the reference had the highest number of covered genes. For experimental verification, we considered using a commercially available strain if the number of covered genes was at most 1% less than the best-fitting strain.

#### Diversity analysis

Diversity analysis of fungal and bacterial communities was performed in R version 3.6.1 using the package vegan [63] version 2.5-5. Testing for significant differences in alpha diversity between time points was performed using a two-sided Wilcoxon signed-rank test. Resulting *p* values were adjusted for multiple testing using FDR. UniFrac metrics measured beta diversity by accounting for phylogenetic similarities of different species. Weighted UniFrac gives the most importance to dominant species. Unweighted UniFrac does not consider abundance. Generalized UniFrac with *α* = 50% gives the most weight to moderately abundant species [64] and the generalized UniFrac with *α* = 75% to species with abundance between median and dominant levels.

### Transcriptional activity

Relative abundances using DNA and RNA were normalized to transcripts per million. Let *f* denote a gene or pathway. The transcriptional activity of *f* is defined as the TPKM-normalized RNA abundance of *f* divided by the TPKM-normalized DNA abundance of *f*.

### Core metatranscriptome

The core metatranscriptome was described in [20]. Briefly, we used MetaCyc pathway relative abundances as generated by HUMANn2 for both DNA and RNA. We calculated transcriptional activity for each pathway. The core metatranscriptome was defined as the set of pathways with a sample prevalence of at least 80% with variable metatranscriptome having prevalence of 30 to 80%. Pathways with less than 30% prevalence were ignored.

### Contributional alpha diversity

We followed the procedure in [26], with some exceptions. For each MetaCyc pathway (PWY), the contribution of species to the pathway was determined. Ecological alpha diversity measures (Shannon and Simpson) were applied per sample and separately using DNA and RNA data. Mean diversity per sample was used to test for significant differences between time points using pairwise two-sided signed Wilcoxon tests. Resulting *p* values were corrected for multiple testing using false discovery rate (FDR).

### Statistics for MGS and ITS abundance

We used MetagenomeSeq [62] version 1.22.0 with a zero-inflated Gaussian mixture model. Following the MetagenomeSeq vignette, CSS normalization was applied on relative abundance data. All possible pairwise tests between the different sampling time points were performed (baseline, DT, EPT, and LPT time points). We controlled for patient-wise differences when possible. For MGS, D2 and D4 were excluded to improve signal quality. A 15% prevalence filter was used for each test independently. Controlling for multiple testing was performed on *p* values using FDR.

### Two-way PERMANOVA testing

Stool samples from the same participant were statistically dependent. To test for significant differences in means of beta diversity between different time points, two-way permutational analyses of variance (PERMANOVA) were performed using “subject id” as covariate and “sample time point” as second independent variable. We performed tests on beta diversity matrices using the function “adonis” as implemented in R package vegan with 9999 permutations. We reported *F* values, *R*^2^, and *p* values for “sample time point”. *P* values from pairwise PERMANOVA tests were corrected for multiple testing using FDR.

### Compositionality tests

We implemented a compositionality test from Palleja et al. [27]. Briefly, we used the test to address the issue of false-positive and false-negative findings in compositional data [65]. We accepted significant findings for a species based on relative abundance only if they would still be significant if other species were removed from the abundance table. Therefore, if one species was removed, the data were total-sum normalized and *p* values calculated. The procedure was repeated for all species. The final *p* value for a species was determined using the highest calculated *p* value. Thus, a species could not become significant because of depletion or inflation of another dominant species. Since this test was very conservative, we used a higher *q* value of 0.1 to decide significance to avoid overlooking potential findings.

### Correlation analyses using stool metabolite abundance

To identify metabolites with a potential effect on *Candida*, *Saccharomyces*, *Penicillium*, and *Aspergillus* spp., we calculated Spearman’s correlations for total-sum scaling (TSS) ITS abundance and both bile acid and MicroMET profiles. To account for zero-inflation, we considered only samples with nonzero abundance of *Candida albicans* (5 samples). We then considered all significant correlations (*p* < 0.05) with an absolute correlation of at least 60%.

To identify direct or indict bacterial producers of the metabolites, total-sum scaled MGS abundances were correlated with log2 transformed metabolites abundances. Correlation was inferred using sparse partial least squared analysis (sPLS) by utilizing relevance vectors (R package mixOmics [30]).

### Co-abundance networks

Co-abundance networks were created based on total-sum-normalized data using BAnOCC [31]. Significance of an edge was determined as described [20]. For posterior inference, we used the 95% credible interval. An edge was therefore considered significant if the corresponding 95% credible interval did not contain zero. Only significant correlations with an absolute estimated coefficient of at least 30% were used for analysis. Significant changes in network structure between any two time points were determined using Wilcoxon signed-rank tests on node degree. Effect sizes are reported in terms of a standardized effect size analogous to the one used for the Mann-Whitney test, $r=z/\sqrt{n}$, where *z* is the *z*-statistic of the paired test and *n* is the number of observations. *r* values are analogous to Pearson correlation coefficients. Hence, *r* ranges from − 1 (100% decrease) to 1 (100% increase). Formula and implementation can be found in the R package “rcompanion”.

#### Fungal species co-abundance network

TSS-normalized operational taxonomic unit (OTU) abundances based on ITS2 data were used. OTUs detected in less than 10% of samples were removed. BAnOCC was executed with 5 chains, 5000 iterations, and 1000 warmup cycles to reach convergence. BAnOCC was used as described above.

#### MGS-ITS network with BAnOCC

MGS and ITS relative abundances were independently total-sum normalized. Only species measured in 25% of samples were used further. Abundances of less prevalent species were summed per sample into a group called “other” to maintain library sizes. MGS and ITS features abundances were combined and analysed using BAnOCC as described above.

#### RNA-PWY-ITS network with BAnOCC

RNA abundances of PWY and ITS were independently total-sum normalized. A 50% samples prevalence filter was applied to make this computation feasible and decrease false-positive rate. Abundances of less prevalent features were summed per sample into a group called “other”. BAnOCC was used as described above.

### Supernatant experiments

#### Strains and culture conditions

*Odoribacter splanchnicus* (DSM20712), *Bacteroides eggerthii* (DSM20697), *C*. *albicans* (SC5314/ ATCC MYA-2876), *C*. *albicans* (ATCC 10231), and *C*. *albicans* (ATCC 18804) were grown at 37 °C under anaerobic conditions (anaerobic gas mixture, 95% N_2_, and 5% H_2_) in pre-reduced modified Gifu anaerobic media (mGAM; Nissui Pharmaceutical Co. Ltd.) broth for liquid cultures or mGAM broth supplemented with agar (Nissui Pharmaceutical Co. Ltd.) for growth on plates.

#### Sterile bacterial supernatants

Bacterial strains grown for 48 h in mGAM broth were subcultured 1:50 in fresh mGAM broth and grown for 48 h in anaerobic conditions at 37 °C. Bacterial cultures were spun down at 11,000×*g* for 5 min. Supernatants were carefully removed and filtered through 0.2-μM syringe filters to remove bacteria in suspension.

#### Supernatant growth inhibition assays

*C*. *albicans* growth rates were analysed in 200 μl liquid mGAM with 50% or 100% sterile bacterial supernatant added. *C*. *albicans* inoculations were at 1:1000 from an overnight culture grown in aerobic conditions at 37 °C. Cultures were in 96-well microtiter plates at 37 °C with orbital shaking 365 cpm (2 mm). Cell densities were measured every 10 min at optical density 600 nm (OD600) using a microtiter reader (BioTek ELx800). Growth rates were calculated by plotting the log OD measurements in log phase and calculating slopes for timepoints in log phase where *r*^2^ was closest to 1, using at least 12 time points (2 h apart).

#### Supernatant metabolite assays

Analysis of SCFA in samples was carried out by MS-Omics as follows. Samples were acidified using hydrochloride acid, and deuterium labelled internal standards where added. All samples were analysed in a randomized order. Analysis was performed using a high polarity column (Zebron™ ZB-FFAP, GC Cap. Column 30 m × 0.25 mm × 0.25 μm) installed in a GC (7890B, Agilent) coupled with a quadropole detector (5977B, Agilent). The system was controlled by ChemStation (Agilent). Raw data was converted to netCDF format using Chemstation (Agilent), before the data was imported and processed in Matlab R2014b (Mathworks, Inc.) using the PARADISe software described by Johnsen et. al [68].

Other compounds such as bile acids were analysed using MS/MS. The analysis was carried out using a Thermo Scientific Vanquish LC coupled to Thermo Q Exactive HF MS. An electrospray ionization interface was used as ionization source. Analysis was performed in negative and positive ionization mode. The UPLC was performed using a slightly modified version of the protocol described by Catalin et al. (UPLC/MS Monitoring of Water-Soluble Vitamin Bs in Cell Culture Media in Minutes, Water Application note 2011, 720004042en). Peak areas were extracted using Compound Discoverer 2.0 (Thermo Scientific). Identification of compounds were performed at four levels: level 1—identification by retention times (compared against in-house authentic standards), accurate mass (with an accepted deviation of 3 ppm), and MS/MS spectra; level 2a—identification by retention times (compared against in-house authentic standards), accurate mass (with an accepted deviation of 3 ppm); level 2b—identification by accurate mass (with an accepted deviation of 3 ppm), and MS/MS spectra; level 3—identification by accurate mass alone (with an accepted deviation of 3 ppm).

### *C*. *albicans* growth inhibition by metabolites

Metabolites were acquired from the companies Sigma-Aldrich, Merck KGaA, and Roth. More specific details can be found in Suppl. Table 15.

#### *C*. *albicans* growth curves

Dilution series of metabolites in water were started at concentrations approximately 10-fold below maximum solubility in water where applicable (Suppl. Table 16). Dilutions were in synthetic SD medium (1× yeast nitrogen base, 2% glucose, 0.5% NH_4_SO_4_). *C*. *albicans* was grown overnight in YPD (1% yeast extract, 2 % peptone, 2 % glucose), washed 3× in sterile water, and inoculated at 1:100 (OD_600_ ≈ 0.2). Absorbance was measured every 15 min with an infinite M200pro microwell plate reader (Tecan, Austria) set to 30 °C with intermittent shaking (10 s orbital shaking before each measurement). Generation times were calculated from the obtained triplicate growth curves.

#### Host cell damage assays

To determine the influence of metabolites on the general capacity of *C*. *albicans* to cause host cell damage, we used an established epithelial cell model based on the vaginal epithelial cell line A431. A431 were grown in RPMI media containing 10% foetal bovine serum (FBS), and 200 μl cells at 10^5^ cells/ml were seeded into 96-well plates and incubated at 37 °C, 5% CO_2_. After 48 h, cells were washed with 1× PBS, and 100 μl compound at indicated concentrations was added, followed by 100 μl *Candida* cells at multiplicity of infection 1. Incubation continued under the same conditions for 24 h. Basal lactate dehydrogenase (LDH) release (low control) was determined with 200 μl RPMI, and maximum LDH release (high control) determined by addition of 100 μl 0.5 % Triton X-100 to cells in 100 μl RPMI. Plate were centrifuged at 250×*g* for 10 min and supernatants were removed and diluted 1:10 and mixed with 100 μl freshly prepared LDH assay mix (Roche). After 25 min at room temperature in the dark, LDH activity was determined with a microplate reader (Tecan infinite M200) as absorbance (A) at 492 nm, with 660 nm as a reference. Damage was calculated as (*A*_sample_ − *A*_low_)/(*A*_high_ − *A*_low_).

#### *C*. *albicans* morphology

The effect of metabolites on *C*. *albicans* morphology was tested at all concentrations used in cell damage assays. Metabolites were diluted in 250 μl RPMI medium with 10% FBS and added to 250 μl *C*. *albicans* in RPMI in 24-well plates to indicated concentrations. Plates were incubated at 37 °C and 5% CO_2_ for 4 h to induce hyphae formation. Medium was removed and cells fixed with Histofix 4% formaldehyde solution. Morphology was evaluated using an inverse microscope (Axio Zeiss Vert. A1) to differentiate yeasts, hyphae, and pseudohyphae.

### Pairwise co-cultivation experiments

Interactions between *C*. *albicans* and *B*. *eggerthii* and *O*. *splanchnicus* were assayed via pairwise cultivations. *C*. *albicans* cell counts were compared to control conditions of cultivation without bacteria.

Fungal and bacterial cells were grown anaerobically at 37 °C for up to 48 h in mGAM and used as inocula for pairwise experiments. Inocula biomasses were estimated via OD600 and adjusted to 1.0 by diluting in appropriate media. Inocula were transferred to microplates containing the same media to a final OD600 of 0.01. Ratios of fungal to bacteria cells were 1:1. Microplates were incubated at 37 °C statically under anaerobic conditions. Cell counts from inocula were resolved, prior to the co-cultivation experiments, via flow cytometry (BD LSRFortessa, BD Biosciences, Franklin Lakes, NJ, USA).

Five microplates were prepared using the same inoculum. Microplates were removed from the anaerobic chamber every 5 h (0, 5, 10, 15, and 20 h cultivation). Cells were immediately fixed in 2% formaldehyde for 15 min at room temperature by mixing an equal amount of sample volume and 4% formaldehyde (Sigma-Aldrich, Saint Louis, MI, USA) [66, 67]. After fixing, total *C*. *albicans* cells were counted via flow cytometry (BD LSRFortessa, BD Biosciences, Franklin Lakes, NJ, USA). Experiments were performed in triplicate.

## Supplementary information

**Additional file 1: Supplementary Figures.**

**Additional file 2.** Statistical results for differences in fungal relative abundance between time points at genus level.

**Additional file 3.** Statistical results for differences in fungal relative abundance between time points at species level.

**Additional file 4.** Fungal network properties and statistical results for differences in node degree between time points.

**Additional file 5.** Growth rate indices of bacterial strains per sample as estimated by GRiD. GRiD values of 1 imply no growth.

**Additional file 6.** Statistical results for differences between group-centroids between different time points using distance-based redundancy analysis.

**Additional file 7.** Information on taxonomic annotation of co-abundant gene clusters (CAG). For each CAG, the bacterial species contributing the highest–and second highest–number of genes is shown. For the most contributing species, the percentage of genes assigned to that species is shown in “PercentageOfTaxonContribution”. The column “trusted” indicates if the corresponding cluster passed our annotation criteria.

**Additional file 8.** Statistical results for differences in MGS relative abundance between time points.

**Additional file 9.** MGS network properties and statistical results for differences in node degree between time points.

**Additional file 10.** Cross-kingdom correlations representing the MGS-ITS correlation network. Correlations are listed for each time point.

**Additional file 11.** List of bacterial species with predicted positive or negative correlation to selected fungal species. The table further contains information on the corresponding MGS, time-related fold-changes, and for which antibiotic drugs the observation was made.

**Additional file 12.** Statistical results for differences in node degree between time points in RNA-MetaCyc network. Results are given for the entire network as well as differences based on funcational categories.

**Additional file 13.** Significant correlations between fungal species and stool metabolites.

**Additional file 14.** Patient information.

**Additional file 15.** List of published genome assemblies available on NCBI. A subset of these were used to infer strain information on several MGS clusters.

**Additional file 16.** Metabolite company information.

**Additional file 17.** Dilution series of metabolites for which we tested their effect on *Candida albicans* growth, morphology, and host cell damage.

## Data Availability

Metagenomic and transcriptomic data generated and analysed during the current study are available in the NCBI SRA repository as Bio Projects PRJNA573821, PRJNA573905, and PRJNA579284. Data from previous work analysed in the current study is available in the NCBI SRA repository as Bio Project PRJINA588313. Metabolite profiles were uploaded to MetaboLights (https://www.ebi.ac.uk/metabolights/) with accession MTBLS1846. Code used for non-standard statistical tests are available as a git repository on https://bitbucket.org/Xentrics/antibiotic-gut-fungi/src/master/.
