# Supplementary material for: Antibiotics create a shift from mutualism to competition in human gut communities with a longer-lasting impact on fungi than bacteria
Source: Microbiome. 2020 Sep 12;8:133. doi: 10.1186/s40168-020-00899-6 (PMC7488854; doi:10.1186/s40168-020-00899-6)
Supplement: Supplementary file 2 — Additional file 1: Supplementary Figures. [file 40168_2020_899_MOESM1_ESM.docx]

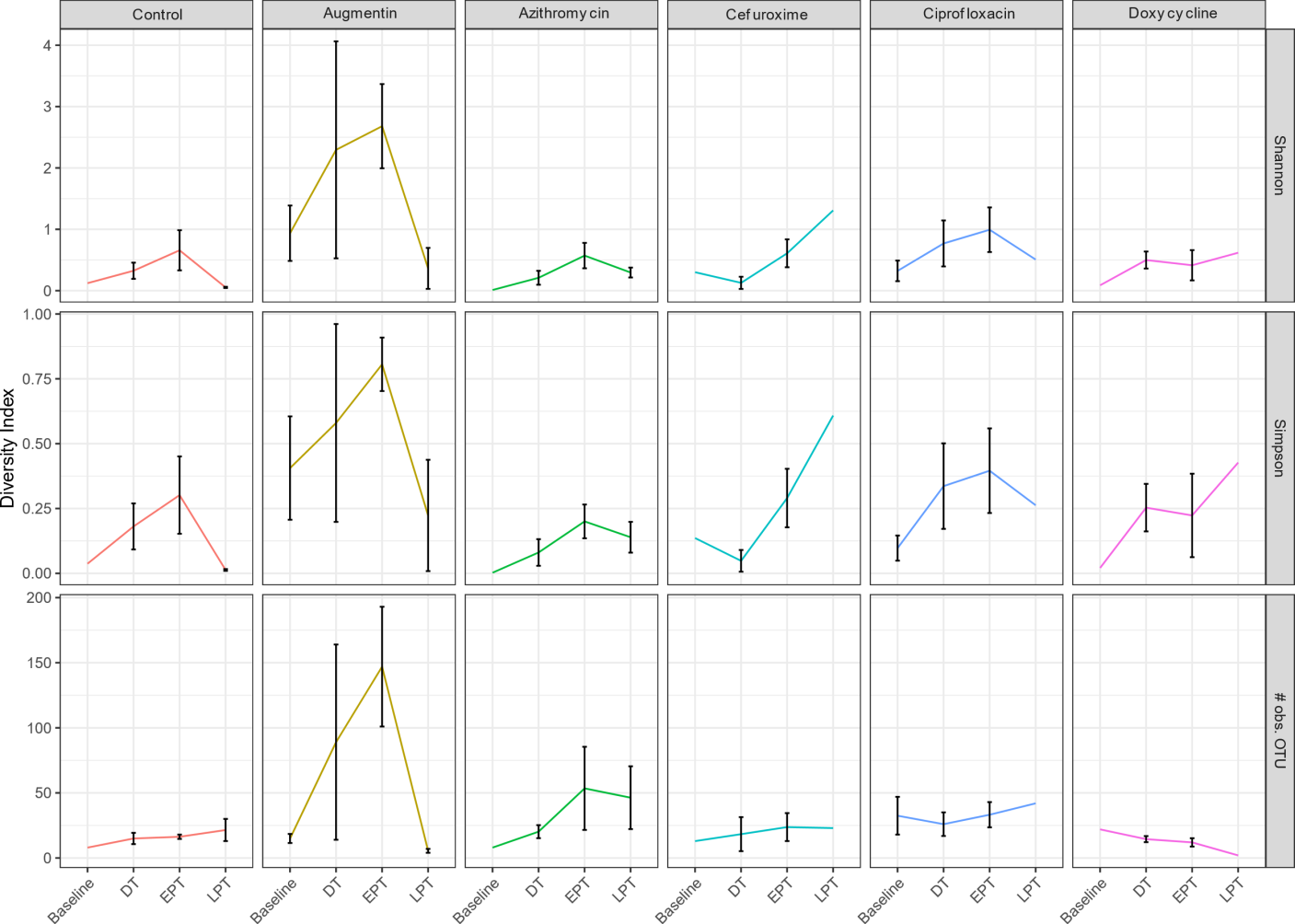


**Suppl. Fig. 1: Fungal species alpha diversity per antibiotic.** Lines show average values. Error lines show standard error. Alpha diversity was measured by Shannon index, Simpson 1-D, and the number of observed OTUs. We focus on Shannon index in the following. Baseline diversity was similar between subjects, ranging from 0.2-0.5 (one outlier with 1.4). In many cases, diversity increased already during treatment (DT). Augmentin showed high variation between subjects and Cefotaxime a slight decrease. In most cases, we observed a spiked increase 30d post treatment (EPT), including controls. Doxycycline exhibited an increase, but overall flatter response. Augmentin showed the strongest peak increase by far. Diversity at 90d post treatment (LPT) was diverse, but indices fell within the range of controls. Overall, Augmentin and Cefotaxime induced a considerable gain in fungal diversity compared to controls. Ciprofloxacin induced higher than control change in one patient as well. Only Azithromycin and Doxycycline showed variation in alpha diversity within the same range as observed for controls.


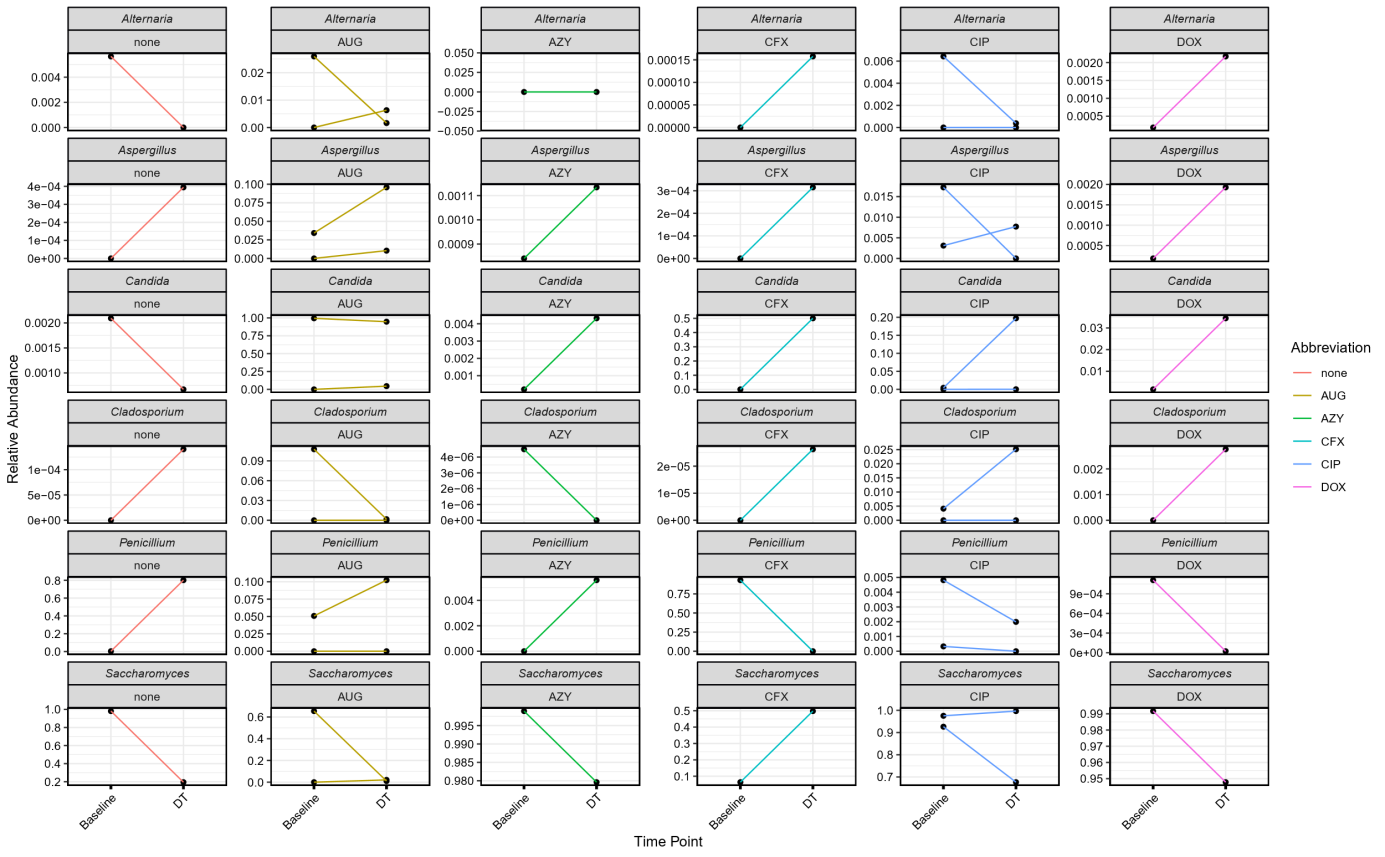


**Suppl. Fig. 2: Mean relative abundance per antibiotic of fungal genera for Baseline and DT.** Only Candida had significant increase in relative abundance (15-fold). At the level of individual antibiotic drugs, we also observed increased relative abundance except for AUG (almost no change).


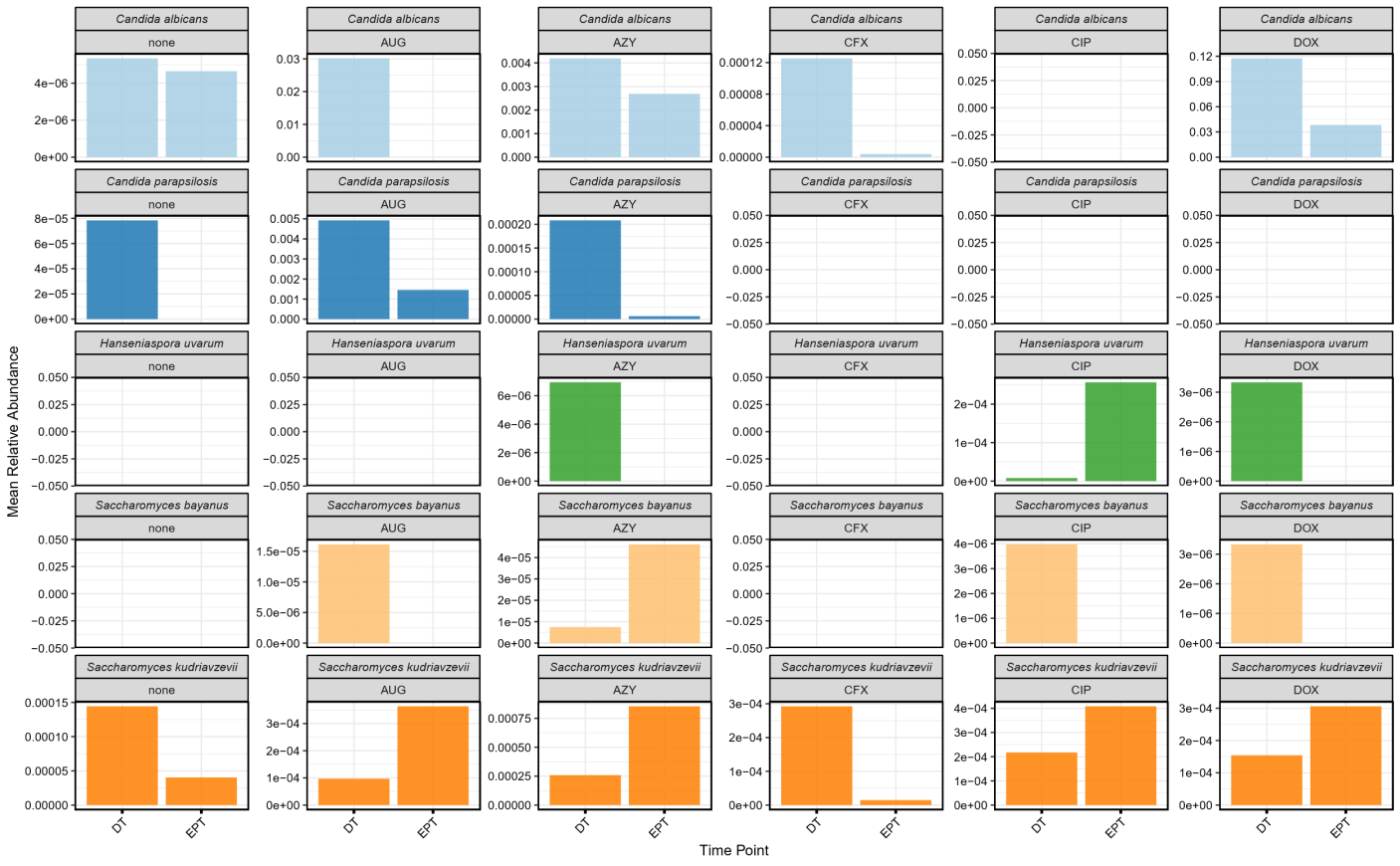


**Suppl. Fig. 3: Mean relative abundance per antibiotic of fungal species with significant change from DT to EPT.** Displayed are five fungal species. *Candida albicans* was measured in each group except in CIP. In the remaining cases, its abundance decreased consistently. AUG and DOX had the strongest effect, especially with respect to the initiate abundance levels > 10%. A minor reduction in growth was also observed in controls, but not as much. *Candida parapsiloses* was measured in at most halve of the patients. In decreased profoundly in relative abundance at EPT in both, treated and untreated patients. The other 3 fungi had less consistent patterns at the level of individual antibiotic drugs.


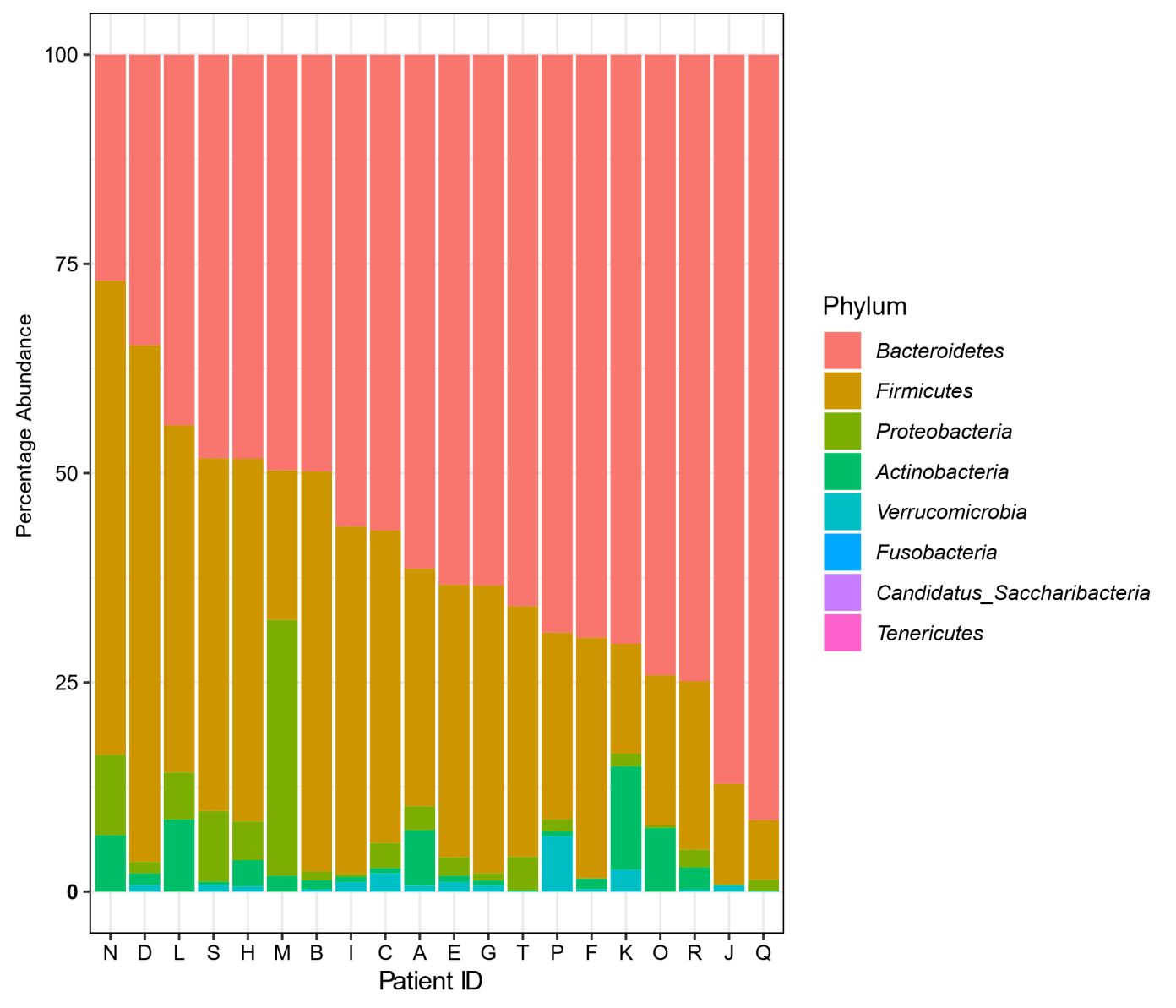


**Suppl. Fig. 4(a): Bacterial phylum composition of baseline samples.** Patients (x-axis) were ordered by the level of the highest contributing phylum (Bacteroides). Phyla were ordered by their average contribution across samples. Most patients were dominated by Bacteroides and Firmicutes spp. These two phyla together accounted for 75-98% of sample-wise abundances. In patient M, we observed an unusual strong contribution by Proteobacteria (30%), which are otherwise the 3rd most abundant phylum on average. Remaining contributions were from Actinobacteria, Verrucomicrobia and Fusobacteria. An insignificant fraction was contributed by Candidatus Saccharibacteria and Tenericutes.


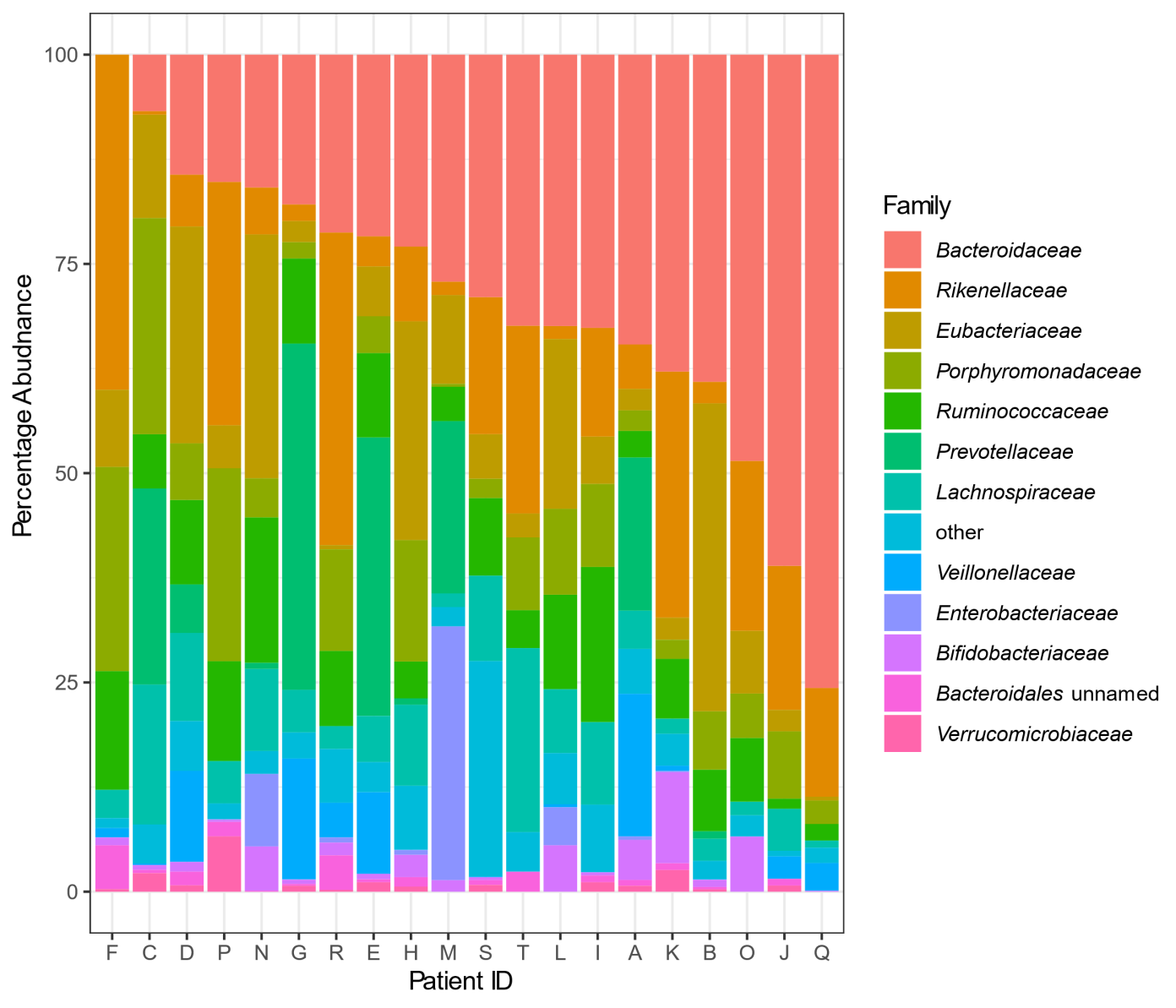


**Suppl. Fig. 4(b): Bacterial family composition of baseline samples.** Patients (x-axis) were ordered by the level of the highest contributing phylum (*Bacteroidaceae*). The 12 most abundant families across samples are shown. Abundance from other families were summed up as “other”. Families were ordered by their average contribution across samples. Overall, we observed a strong difference between *Bacteroidaceae* spp. (from <1% to 75%) and the remaining bacterial spp. contributions. *Bacteroidaceae, Rikenellaceae, Eubacteriaceae, Porphyromonoadaceae, Ruminococcaceae, Prevotellaceae* and *Lachnospiraceae* accounted for most contributions across samples. *Enterobacteriaceae* accounted for only 0.02% on average, except patients M (30%), L (5%) and N (9%), which are all control patients.

**
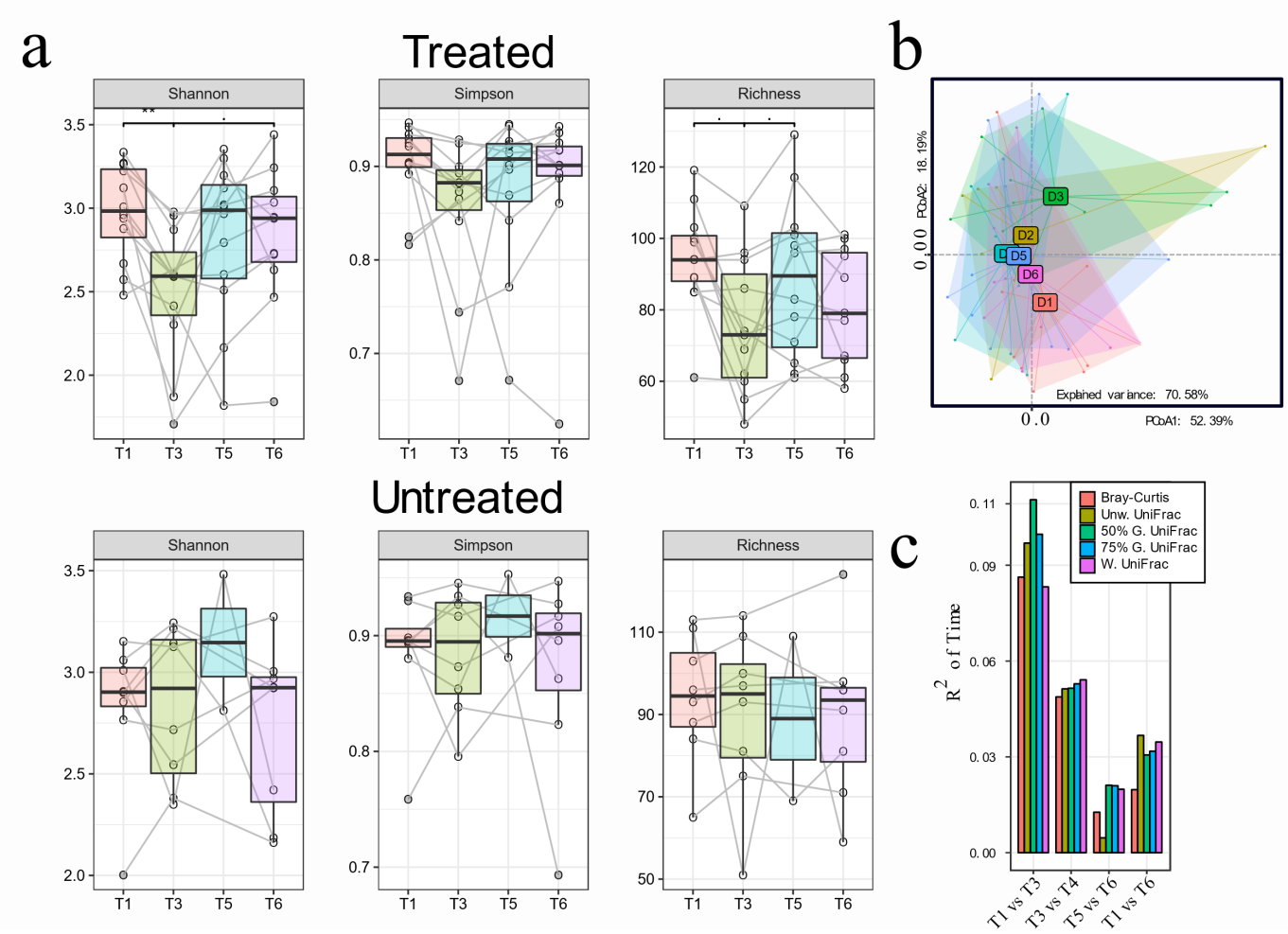
Suppl. Fig. 5: Antibiotics induced severe changes in bacterial community 6 days after treatment. (a-c)** Diversity analysis of samples from subjects using MetaPhlAn2 relative abundances. **(a)** Boxplots showing Species Richness (right), Shannon (left) and Gini-Simpson Index (middle). First row shows diversity for antibiotic treated samples. Second row show alpha diversity for control samples. The median (centerlines), first and third quartiles (box limits) and 1.5x interquartile range (whiskers) are shown. Lines between boxes connect same-donor samples. Statistical testing was performed using a Wilcoxon signed-rank test and p values were adjusted for multiple testing using FDR (q). Signs indicates significance level (**: q<0.01; .: 0.05<q<0.1). **(b)** Principle coordinate analysis of generalized UniFrac distance (a = 0.5) as a measure of beta diversity. **(b-c)** We tested for differences between times in treated subjects while controlling for subjects using a pairwise two-way PERMANOVA. **(c)** R² values of covariate “Time” from pairwise PERMANOVA. Five different measures of beta diversity were tested independently. R² values for consecutive samples are shown.


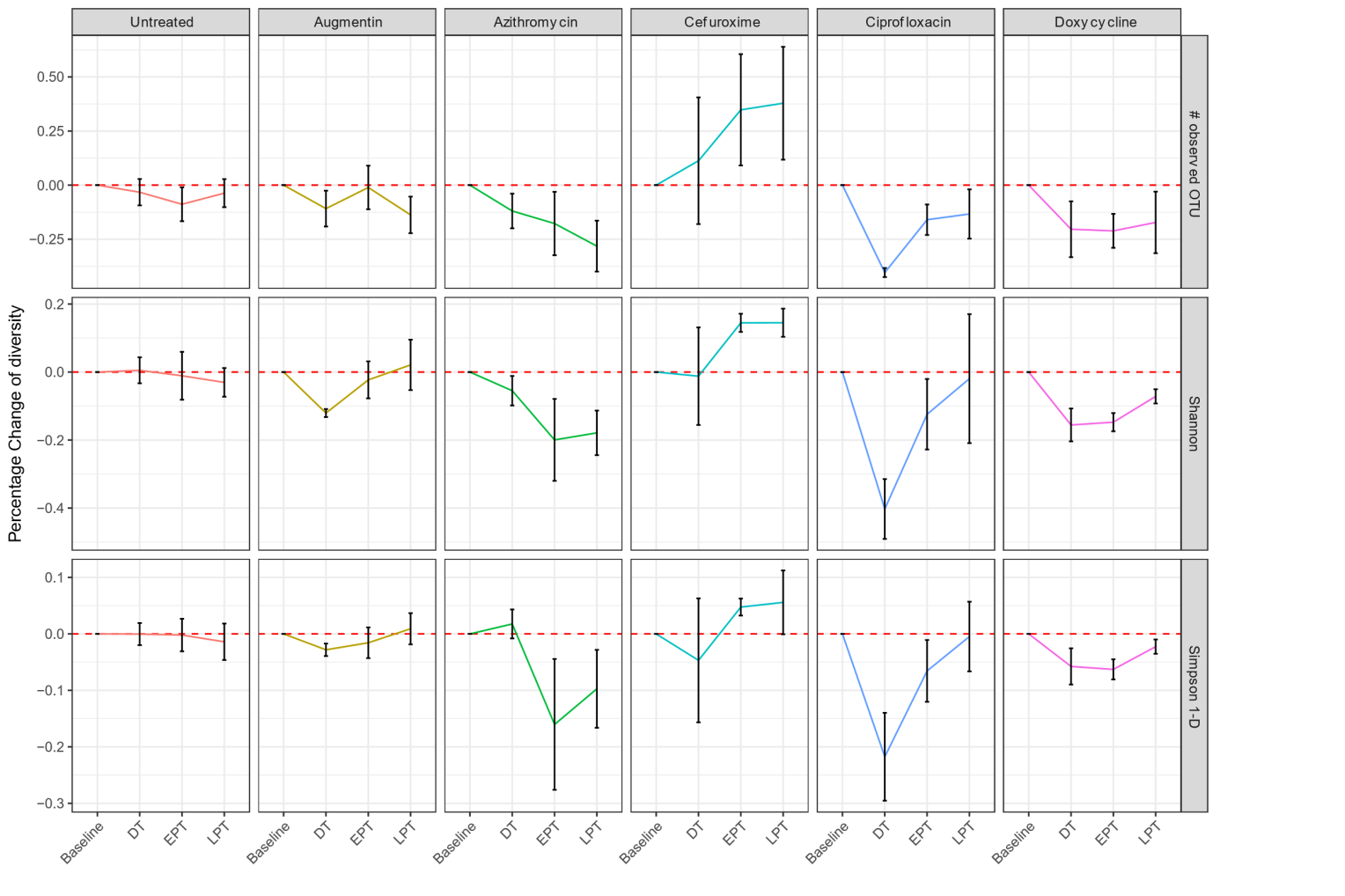


**Suppl. Fig. 6: Proportional change of bacterial species alpha diversity compared to Baseline.** Proportional change of bacterial alpha diversity at species level based on MetaPhlAn2 OTU profiling. In untreated patients, we observed a drop in species richness during after treatment. However, Shannon and Simpson diversity remained mostly unchanged, implying changes due to sequencing depth or other technical artifacts. In antibiotic treated patients, alpha diversity changes the most for Shannon diversity. It decreased by 5%-40% during treatment, except for CFX treated patients. One CFX treated patient showed a monotonous increase in diversity compared to baseline. The strongest, negative impact was observed using CIP. In most patients, alpha diversity was increased 90d post treatment compared to their respective DT and EPT time points. This implies that most of the original diversity was regained after treatment, but not all. Furthermore, the difference between LPT and Baseline alpha diversity range from +18% to -21%. Some of difference in late-post treatment diversity might be explained with natural variation we observed in controls (left-most panel; roughly +/- 5%).


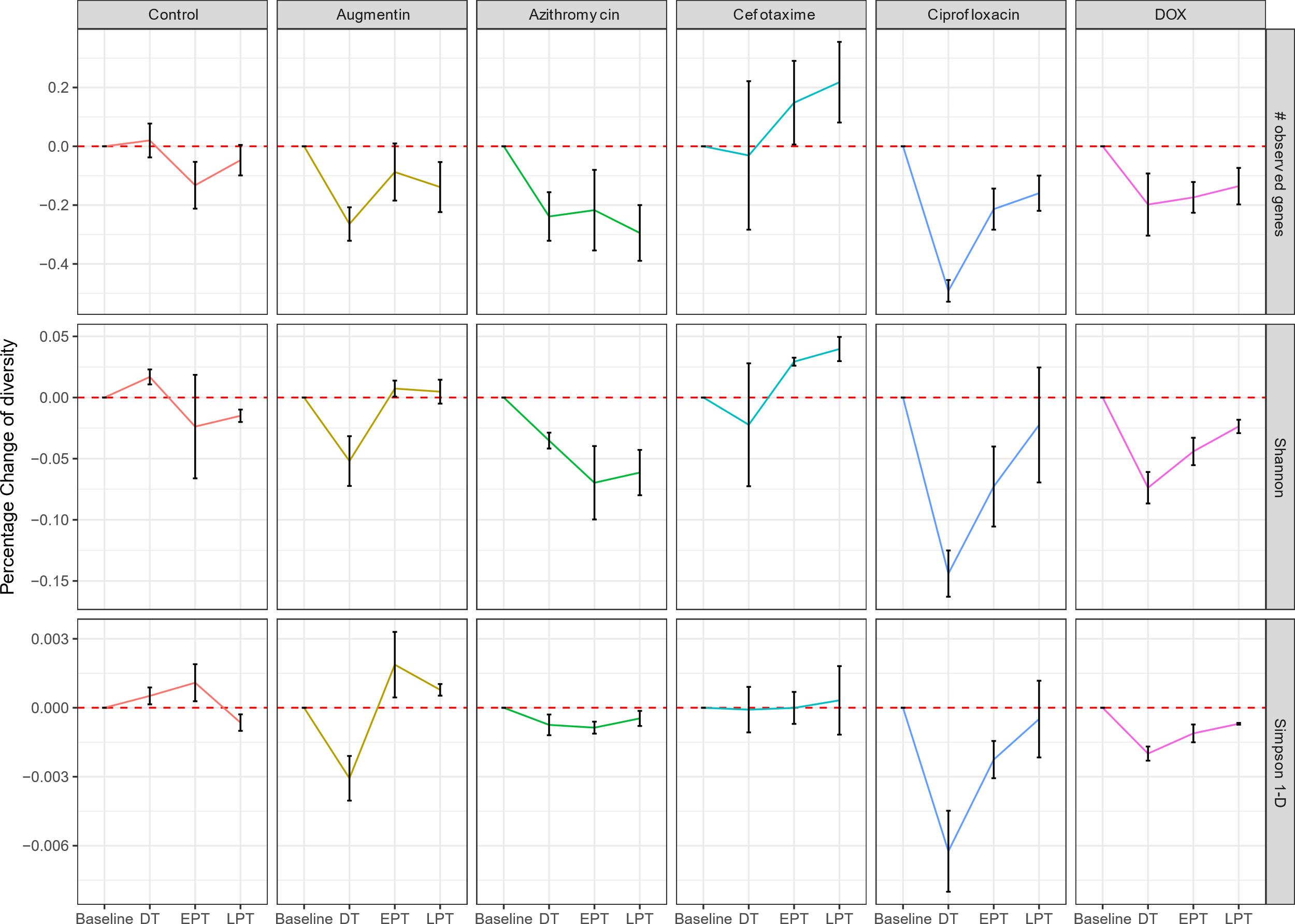


**Suppl. Fig. 7: Proportional change of DNA gene family alpha diversity compared to Baseline.** Gene family abundance was estimated by HUMAnN2 pipeline. In untreated patients, we observed a drop in gene richness after treatment (EPT). However, Shannon and Simpson had contrasting differences to that (mixed for Shannon, increase for Simpson), implying changes due to sequencing depth or other technical artifacts. In antibiotic treated patients, richness decreased by 19%-49% during treatment, except for CFX treated patients. One CFX treated patient showed a monotonous increase in diversity compared to baseline. Differences in Shannon diversity were less severe, ranging from 0.02% to 0.18%. These two findings together imply that many genes lost during treatment (as measured by richness) were in relatively low abundance (and hence did not affect Shannon diversity much). So overall, the changes observed for bacterial species diversity were qualitatively like those for gene family diversity. The major exceptions were the control samples, which exhibited bigger variance in diversity at the functional level compared to the species level.


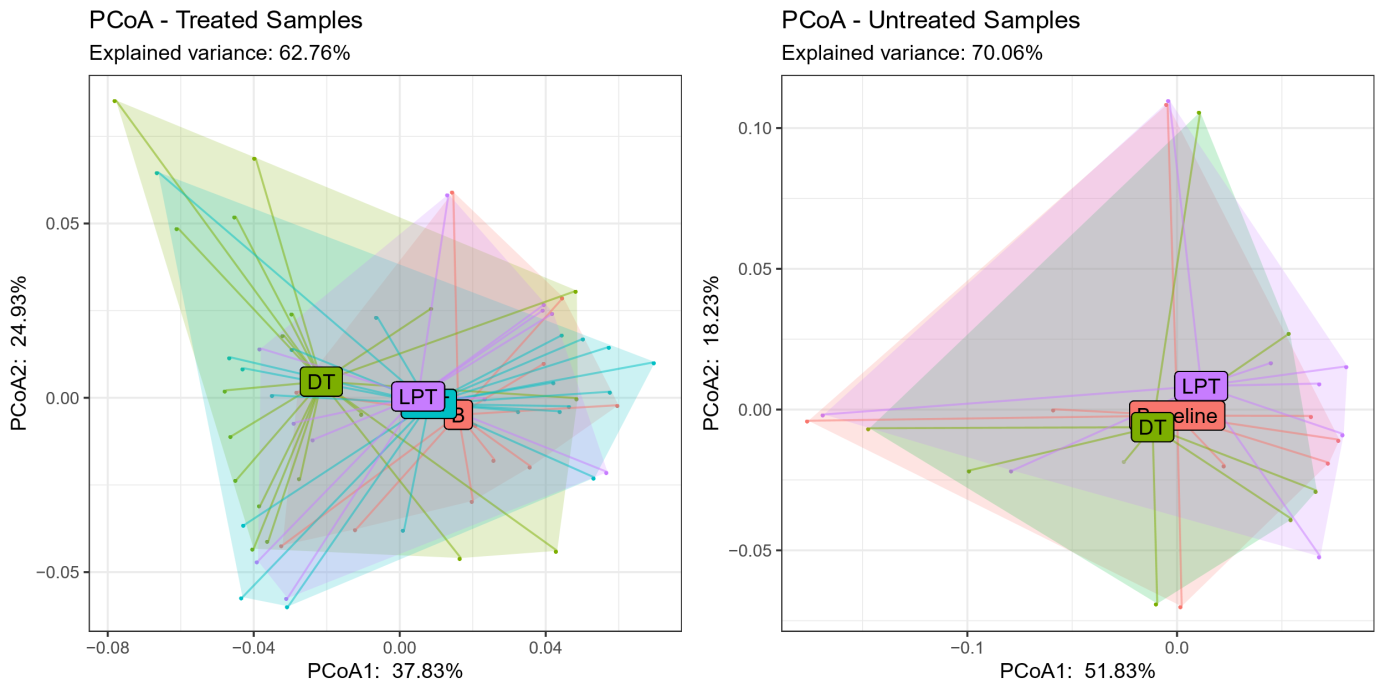


**Suppl. Fig. 8: Principle coordinate analysis of DNA bacterial function beta diversity between time points.** Beta-diversity was measured as Bray-Curtis index. We separate between treated samples (left) and untreated controls (right). Time points were pre-treatment (Baseline, B; red), treatment (DT; green), 30d post treatment (EPT; blue) and 90d post treatment (LPT; violette). In treated samples, we observe a significant difference between Baseline and DT, q=0.014). Control samples did not show significance differences across time points.

**Suppl. Fig. 9: Core- and Variable Metatranscriptome of MetaCyc pathways (PWY).** **(a)** Up-regulated core with RNA/DNA > 1 and prevalence >= 80%. **(b)** Down-regulated core with RNA/DNA < 1 and prevalence >= 80%. **(c)** Variable metatranscriptome with prevalence between 30% and 80%, ordered by mean RNA/DNA ratio. There were no significant differences in RNA/DNA ratio of PWYs between time points.
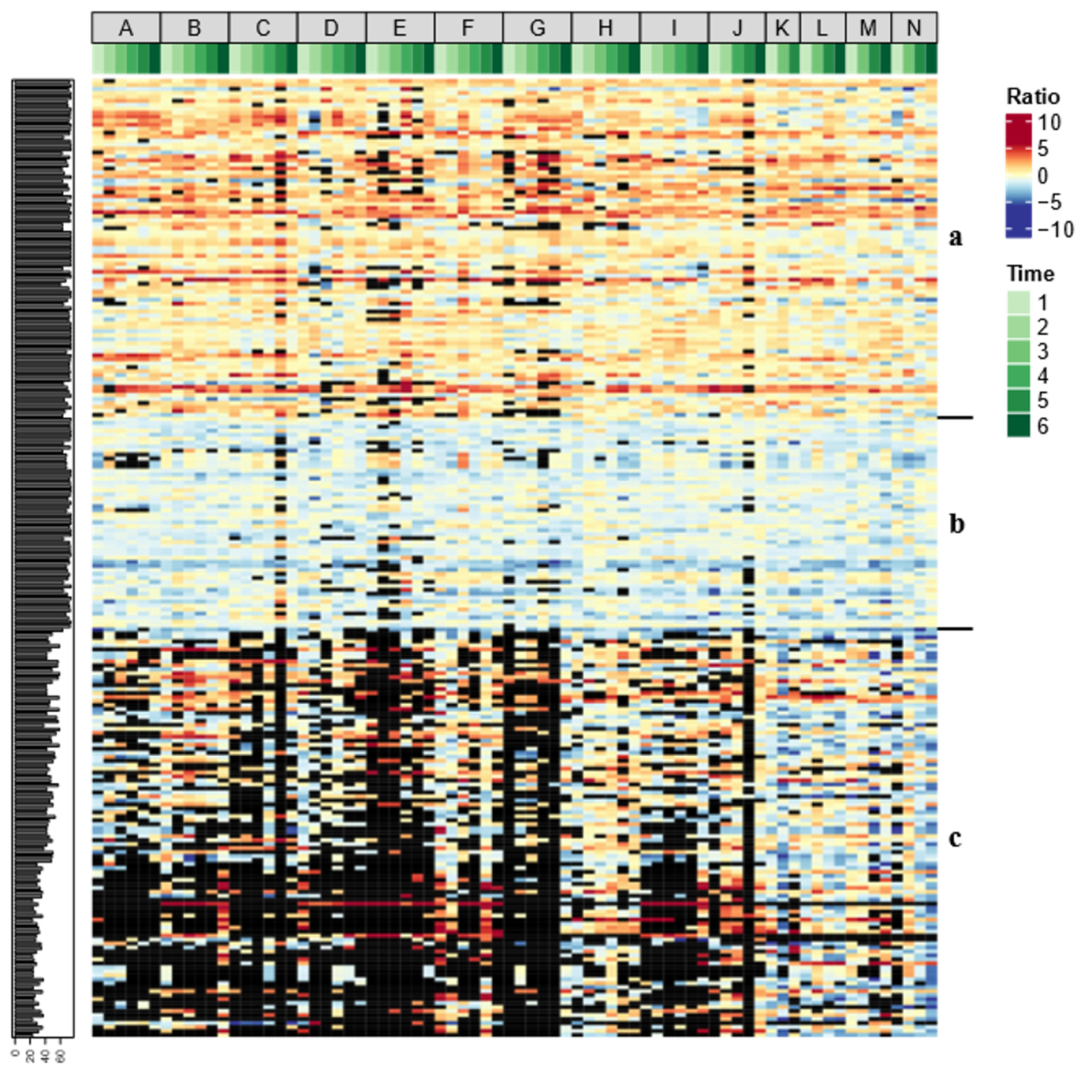


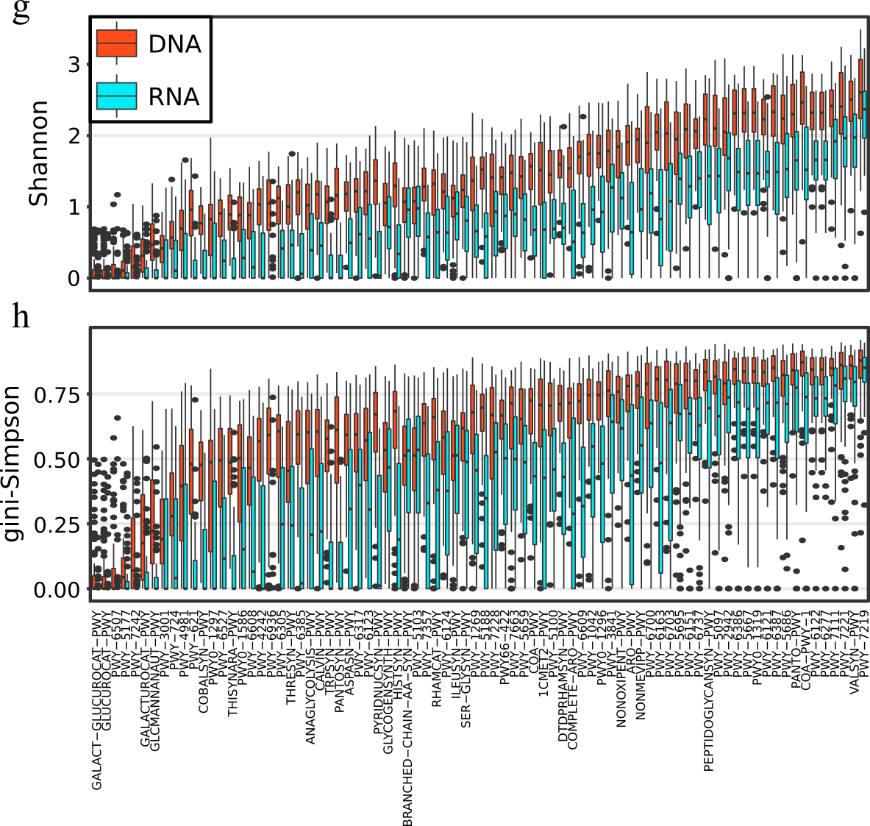
**Suppl. Fig. 10:** Contributional alpha diversity of MetaCyc pathways using Simpson (**g**; 1-D) and Shannon (**h**) diversity indices. Pathways are ordered by the sum of the mean DNA and mean RNA Shannon diversity. RNA diversity was generally lower than DNA diversity. Shannon diversity dropped more gradual compared to Simpson. A Shannon diversity of 2 implies that only 4 species are truly relevant for the contribution to the corresponding pathway. A Simpson diversity of 75% implies that (in addition) 1 species accounts for 75%.

**Suppl. Fig. 11: 26 MGS were sign. diff. abundant during treatment.** Heatmap of z-transformed, cumulative sum scaled relative abundances of significantly differentially abundant MGS between baseline and treatment time (ZIG model; Time and Patient as covariate; q_Time_ < 0.05).


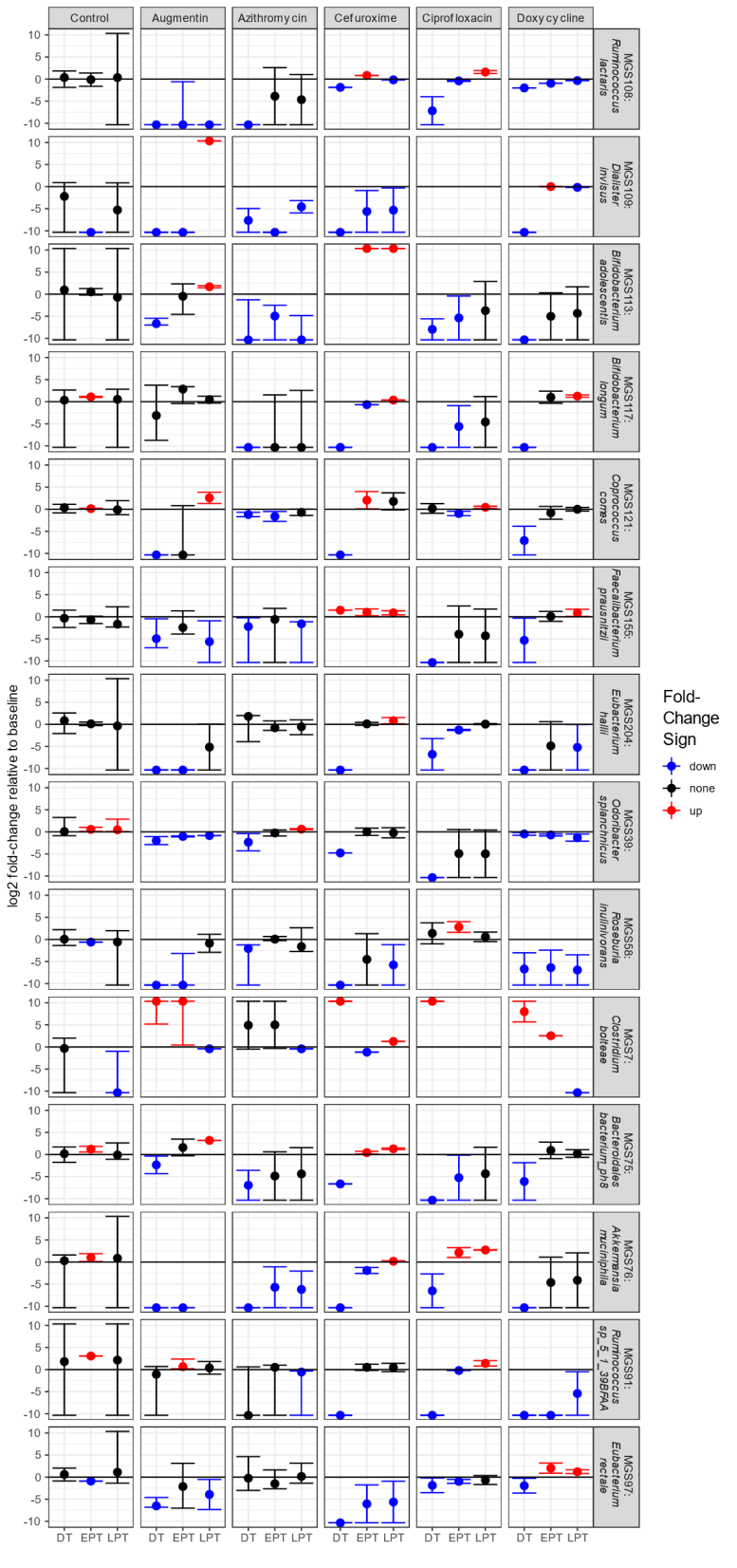


**Suppl. Fig. 12: Fold-change of named MGS.** Out of 26 MGS with sign. change during treatment, 14 had species level annotation. Here, log_2_ fold-changes relative to baseline levels are shown per treatment group. Observation with 0 counts before and after treatment were ignored. Fold-changes of +/- infinity were set to 110% of the strongest non-infinite value to presence/absent observations. Whiskers show minimum and maximum values. Colours indicate if fold-changes were positive (red), negative (blue) or both (black) at a given time in each treatment group.
6 of these were consistently decreased independent of the antibiotic drug used: *Ruminococcus lactaris, Dialister invisus, Odoribacter splanchnicus, Bacteroidetes bacterium ph8, Akkermansia muciniphila, Bifidobacterium adolescentis*.
Among the other species (8), 7 had negative fold change in 4 out of 5 drugs: *Bifidobacterium longum, Coprococcus comes, F. prausnitzii A2-165, Eubacterium hallii, Roseburia inulinivorans, Ruminococcus sp. 5_1_39BFAA, Eubacterium rectale*.
*Clostridium bolteae* had positive fold change in 4 out of 5 drugs.


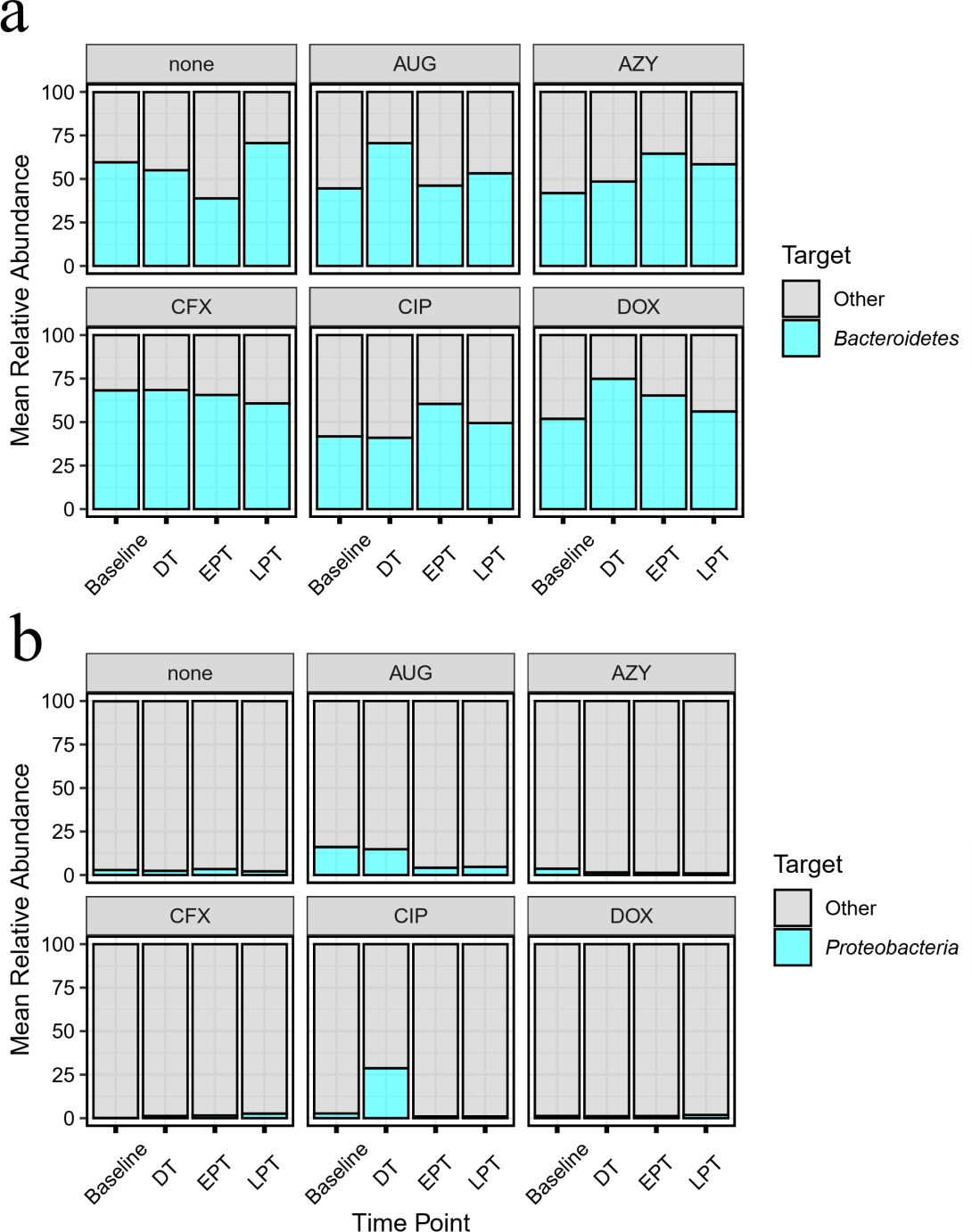


**Suppl. Fig. 13: Mean relative abundance of species with possible differential effect by specific antibiotic classes.** Maier et al. (https://doi.org/10.1101/2020.01.09.893560) reported that broad-spectrum antibiotics targeted all measured - the 40 most common - gut bacteria. However, beta-lactams may have a different influence on *Bacteroides* (a) compared to other species. Here, this would apply to Augmentin (AUG) and Cefuroxime (CFX). In all treated samples, *Bacteroidetes* increased in relative proportion during (DT) or early-post treatment (EPT). Despite the expectation, the effect seems to be rather low in CFX treated samples, which had comparatively high levels of *Bacteroidetes* at baseline. Likewise, Doxycycline (tetracycline class) showed an effect equal to Augmentin. So overall, we only noticed a slight delay in response for CIP and AZY, but not qualitative difference.
Analogous, macrolides may have a different influence on *Proteobacteria*. Here, this would apply to Azithromycin. In most subjects, *Proteobacteria* have less than 1% contribution, making it hard to assess if relative abundance changes are due to colonization difficulties or antibiotic effect. We noticed that Augmentin lead to a severe decline in abundance for this phylum even though it belongs to an entirely different class. In contrast, CIP lead to an increase. Since we have relative abundance, we cannot assess if abundance increase relates to an actual increase in abundance. An increase can be the result of a severe decrease of the remaining community. Such could be the case for CIP.
So all together, we cannot find a selective difference in targets by the different antibiotics used.


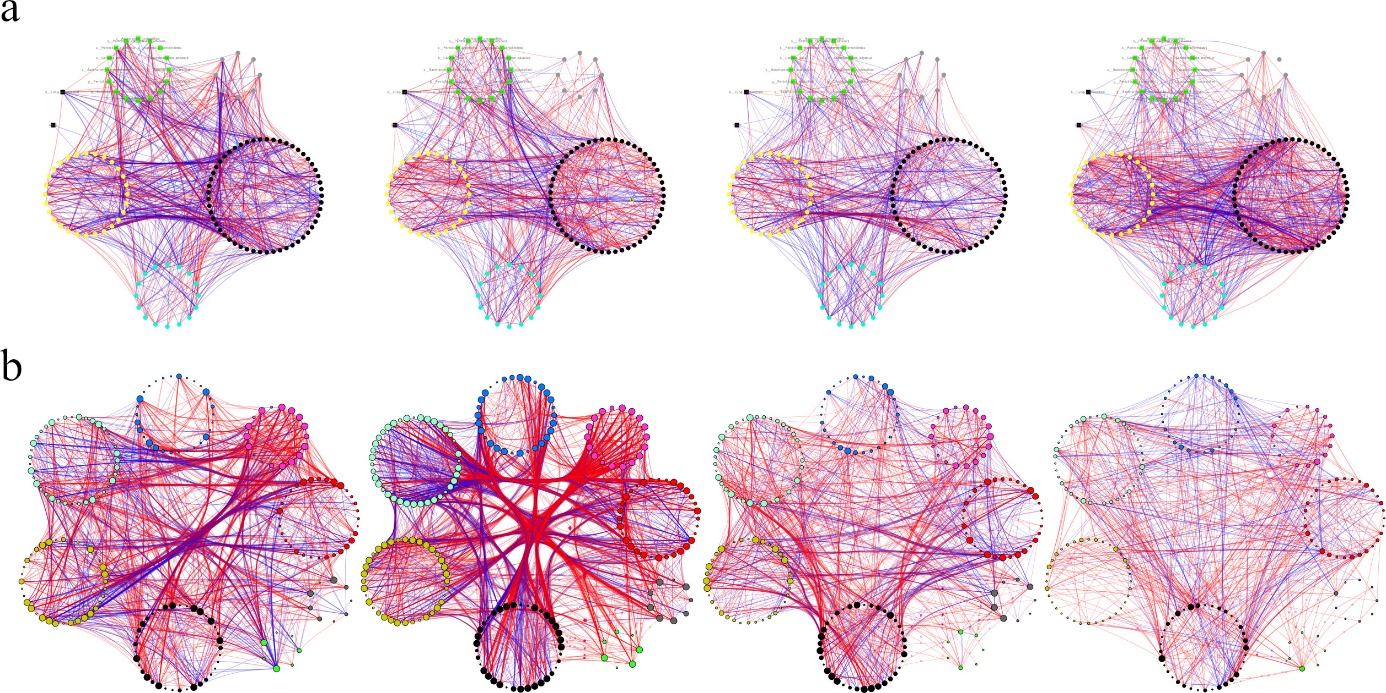


**Suppl. Fig. 14:** **interactions between fungi, bacteria species and pathway expression.** (**a-b**) Co-abundance networks of different time points using BAnOCC with (a) 25% and (**b**) 50% prevalence filter. Only significant edges (based on 95% credibility interval) with |r| >= 0.3 are shown. Negative correlations (blue), positive correlations (red). Networks are ordered from left (Baseline) to right (Late-Post-Treatment). (**a**) Correlations between fungal and bacterial species based on MGS and ITS relative abundances. Node colors indicate phyla. Unclassified MGS are black. (**b**) Correlations between fungal species and pathway expression based on HUMAnN2 RNA pathway and ITS relative abundances. Node colors indicate fungi (green) and functional groups. Superpathways and other pathways which did not fit into the six major categories were grouped into “other”.


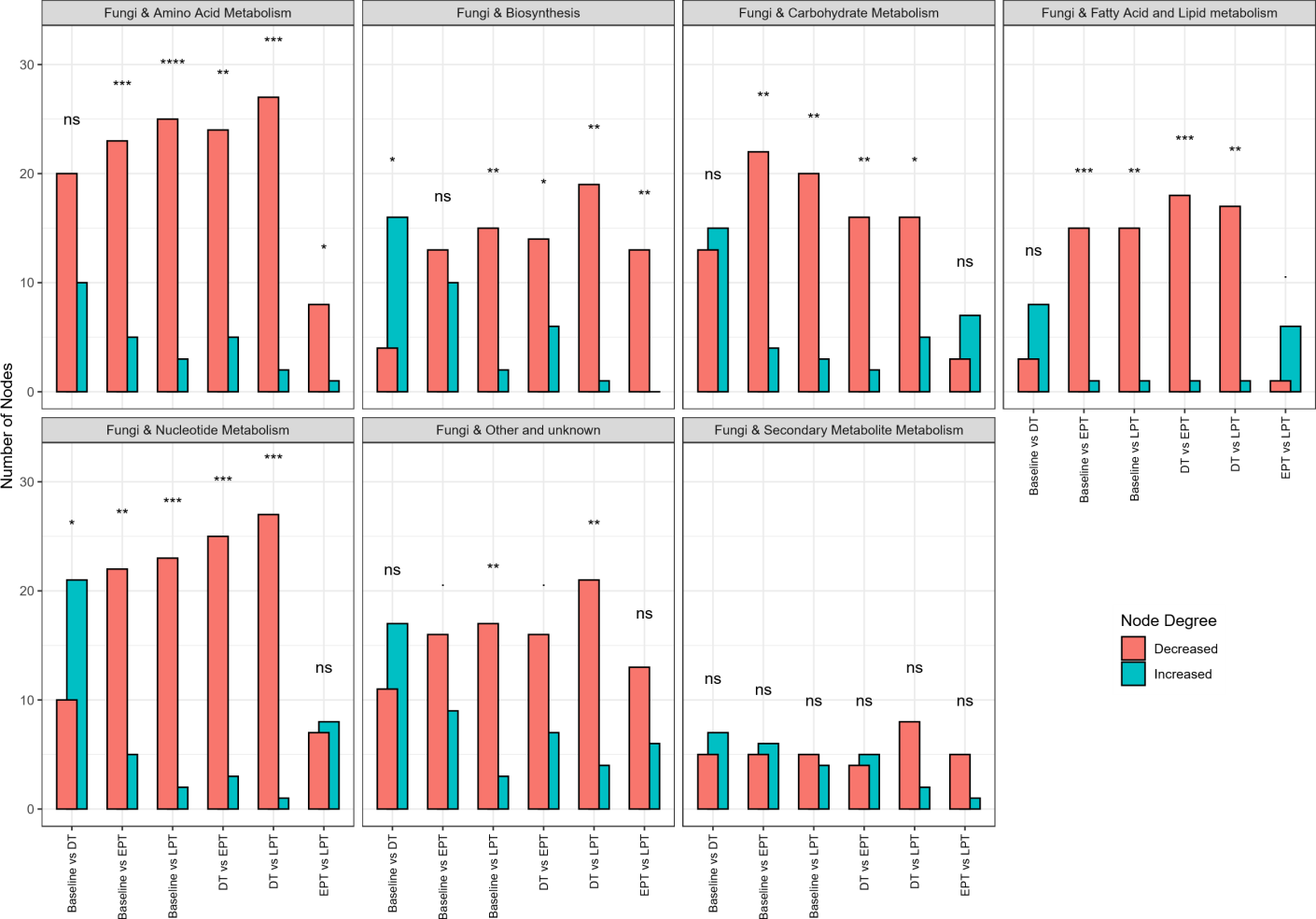


**Suppl. Fig. 15:** Node degree centrality of RNA-PWY-ITS network based on correlations between fungal species and MetaCyc pathway groups. Bar plots showing the number of nodes which increased and decreased in centrality between time points. Statistical testing for significant changes in centrality was performed using a two-sided Wilcox signed-rank test. P values were adjusted for multiple testing. Significance is indicated by symbols (ns: q ≥ 0.05; *: q < 0.05; **: q < 0.01; ***: q < 1e-3; ****: q < 1e-4; *****: q <1e-5).


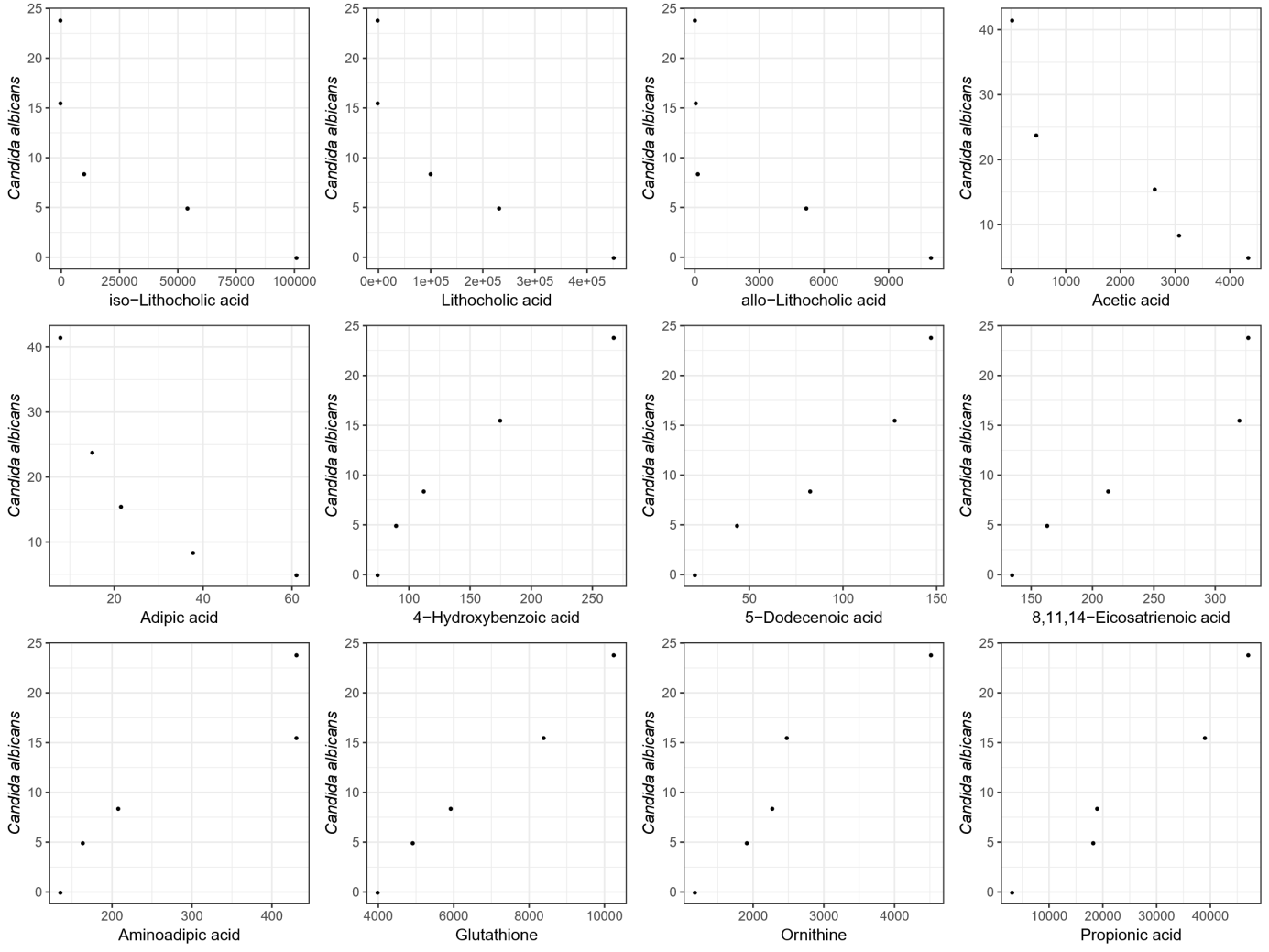


**Suppl. Fig. 16:** Metabolites significantly correlated with C. albicans OTU abundance levels (Spearman |rho| > 0.3; p < 0.05).

**
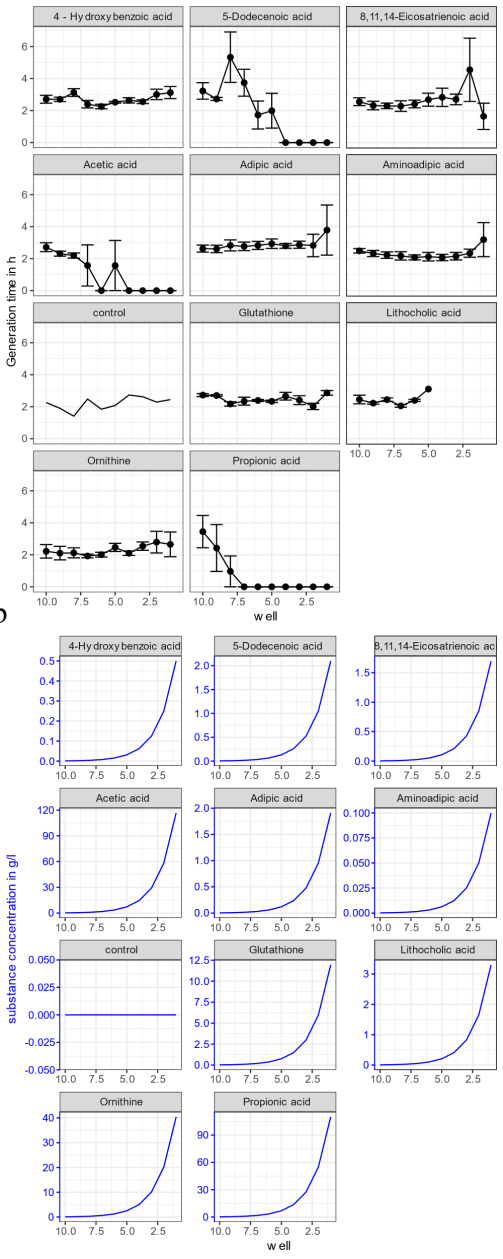
Suppl. Fig. 17: *C. albicans* growth inhibition by metabolites.** (a) Generation times of C. albicans in h over varying degrees of substance concentrations. Substances were diluted (from left to right). For some compounds, measurements failed at higher concentrations. (b) Substance concentration per well in g/l.

**Suppl. Fig. 18: Inhibition of C. albicans growth and host cell damage.** We tested the effect of the metabolites on a human vaginal cell line (A431) at two concentrations each: the lowest concentration where C. albicans growth was significantly inhibited and the highest without any significant effect on growth (table 1; top-left; bottom-left). There was some limited cytotoxicity observed for lithocholic acid and cis-5-dodecenoic acid, but all other substances did not elicit any detectable host cell damage. Glutathione, however, interfered with our assay and was therefore excluded from these studies. Next, we assayed the effect of the same concentration on cell damage by C. albicans. None of the substances affected the host-pathogen interactions at the lower concentration (top-right). The notable exception is cis-5-dodecenoic acid, with severely reduced the damage by C. albicans to the host cells, albeit not at a statistically significant level. At the higher concentrations (bottom-right), where fungal growth was reduced in vitro, we also observed lower host cell damage in presence of the short-chain fatty acids propionic and acetic acid, where damage by C. albicans was nearly fully abolished, and again of cis-5-docenoic acid. To lesser extent and not statistically significant there was a tendency to lower damage also with ornithine (p=0.087) and benzoic acid (p=0.051) in the medium.

**Suppl. Table 1**

| [µg/µl] | Lithocholic Acid | Benzoic Acid | cis-5-Dodecenoic Acid | 8,11,24-Eicosa- trienoic Acid | Aminoadipic Acid | Propionic Acid | Ornithine | Acetic Acid | Adipic Acid |
| --- | --- | --- | --- | --- | --- | --- | --- | --- | --- |
| Subinhibitory | 0.103 | 0.25 | 0.263 | 0.85 | 0.05 | 0.0004 | 1.27 | 0.0004 | 0.95 |
| Inhibitory | 0.413 | 1.00 | 1.05 | 3.40 | 0.20 | 1.70 | 5.05 | 7.29 | 3.82 |

**
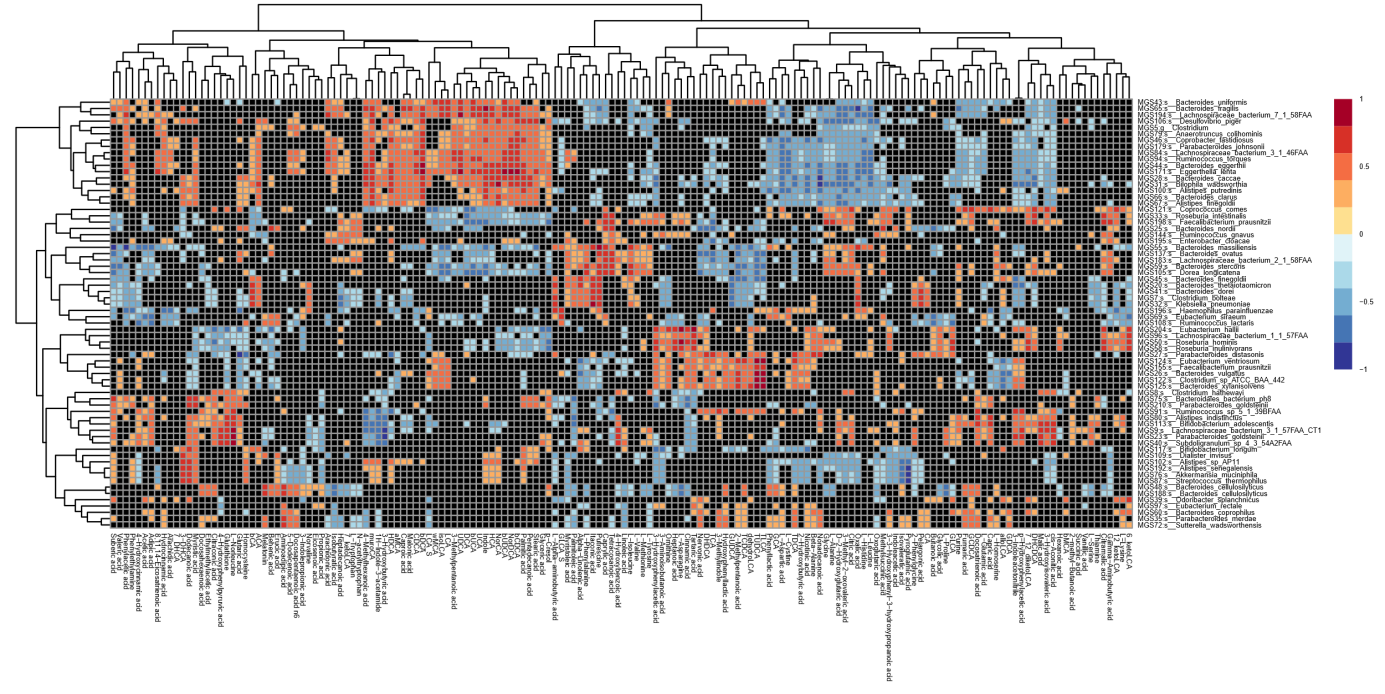
**


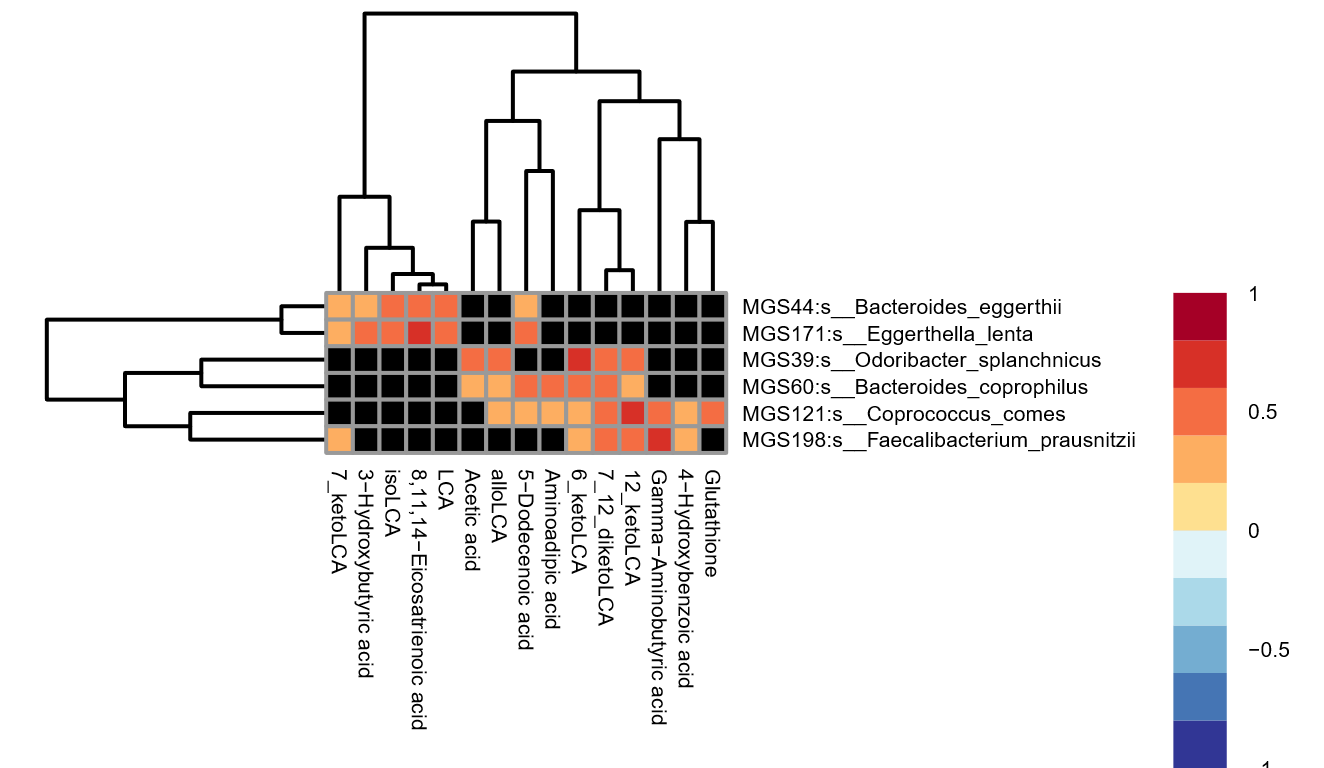


**Suppl. Fig. 19: Correlations of bile acid and metabolite abundance with MGS abundance.** Cell colour indicates correlation strength (blue: negative; red: positive). Absolute correlation below 0.3 and insignificant correlations (p > 0.05) are coloured white.


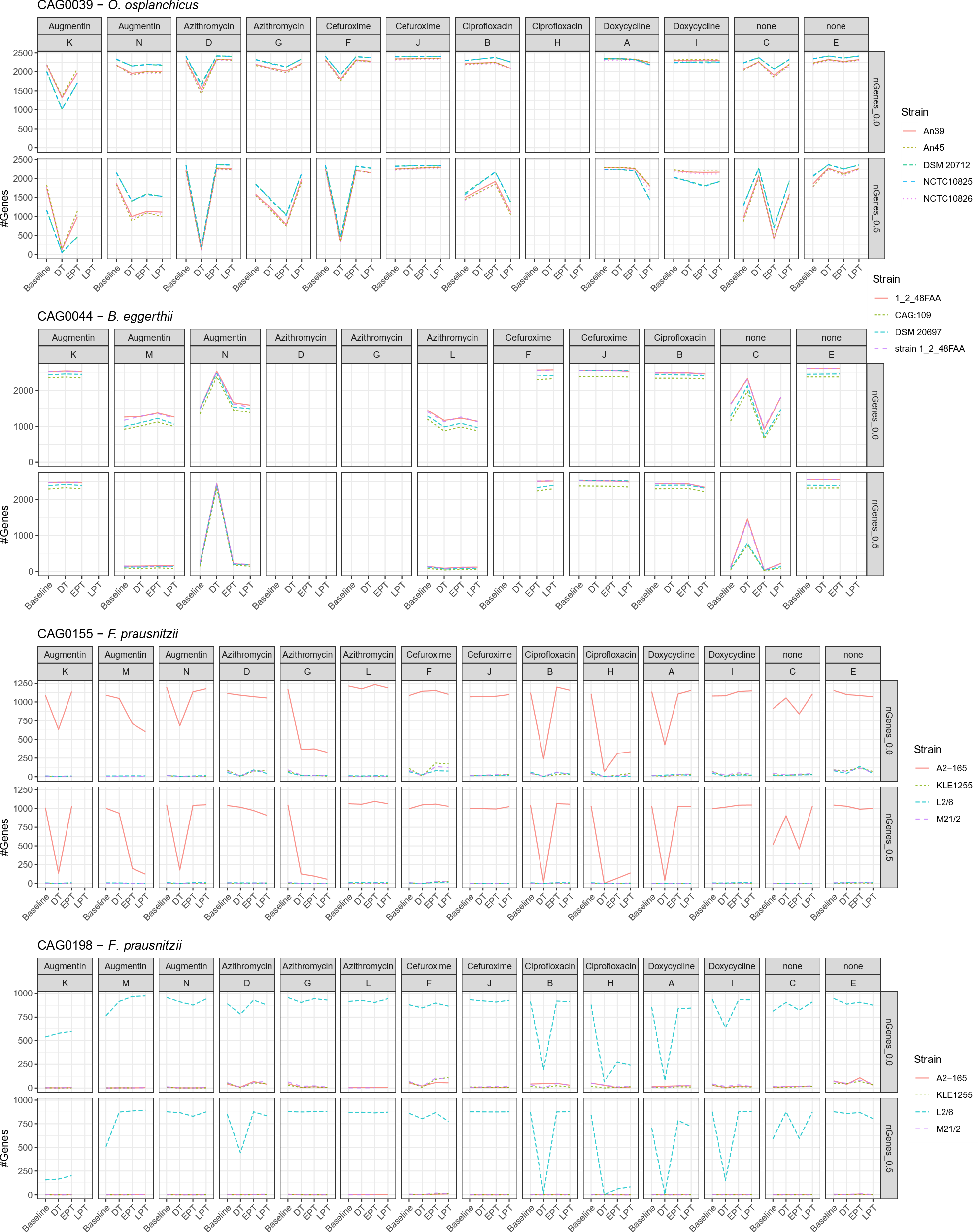


**Suppl. Fig. 20: Number of genes contributing to each MGS.** For each sample, the number of genes with at least 1 gene (first row of each panel) and at least 50% coverage (2. Row) per strain. Different strains are indicated by colour and line type. For Faecalibacterium prausnitzii, we have 2 MGS with exactly 1 matching strain for each (CAG0155 with A2-165 and CAG0198 with L2/6)


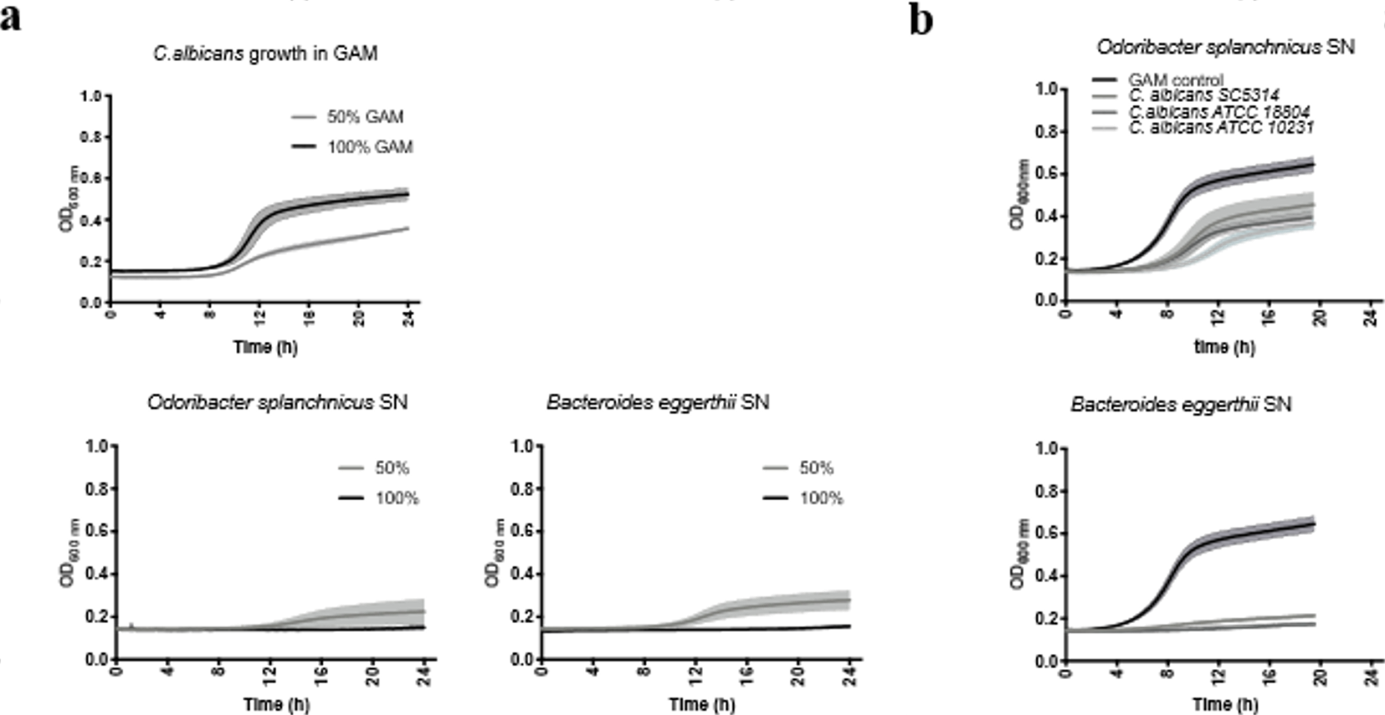
**Suppl. Fig. 21: *C. albcians* growth rate on bacterial supernatant.** **(a)** Growth curves for *C.albicans* strain SC5314 mGAM media with 50% or 100% sterile bacterial supernatant added. **(b)** Growth curves for three *C.albicans* strains; SC5314, ATCC10231 and ATCC 18804 in mGAM media with 100% sterile bacterial supernatant added.

**
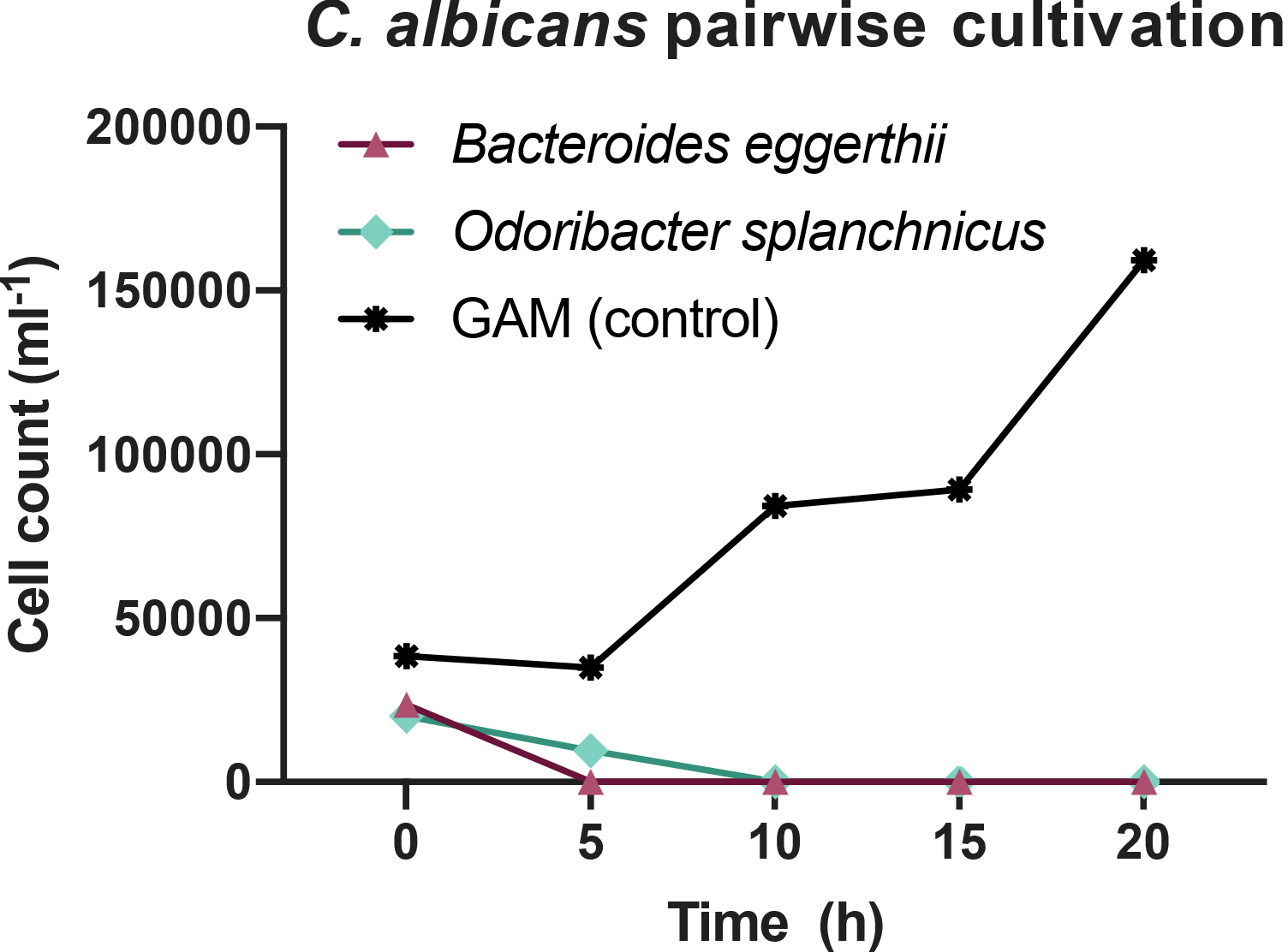
**

**Suppl. Fig. 22: Co-cultivation of *C. albicans* with bacterial species.** Cell counts of *C. albicans* was measured using FACS. When co-cultured with B. eggerthii or O. splanchnicus, no growth occurred.


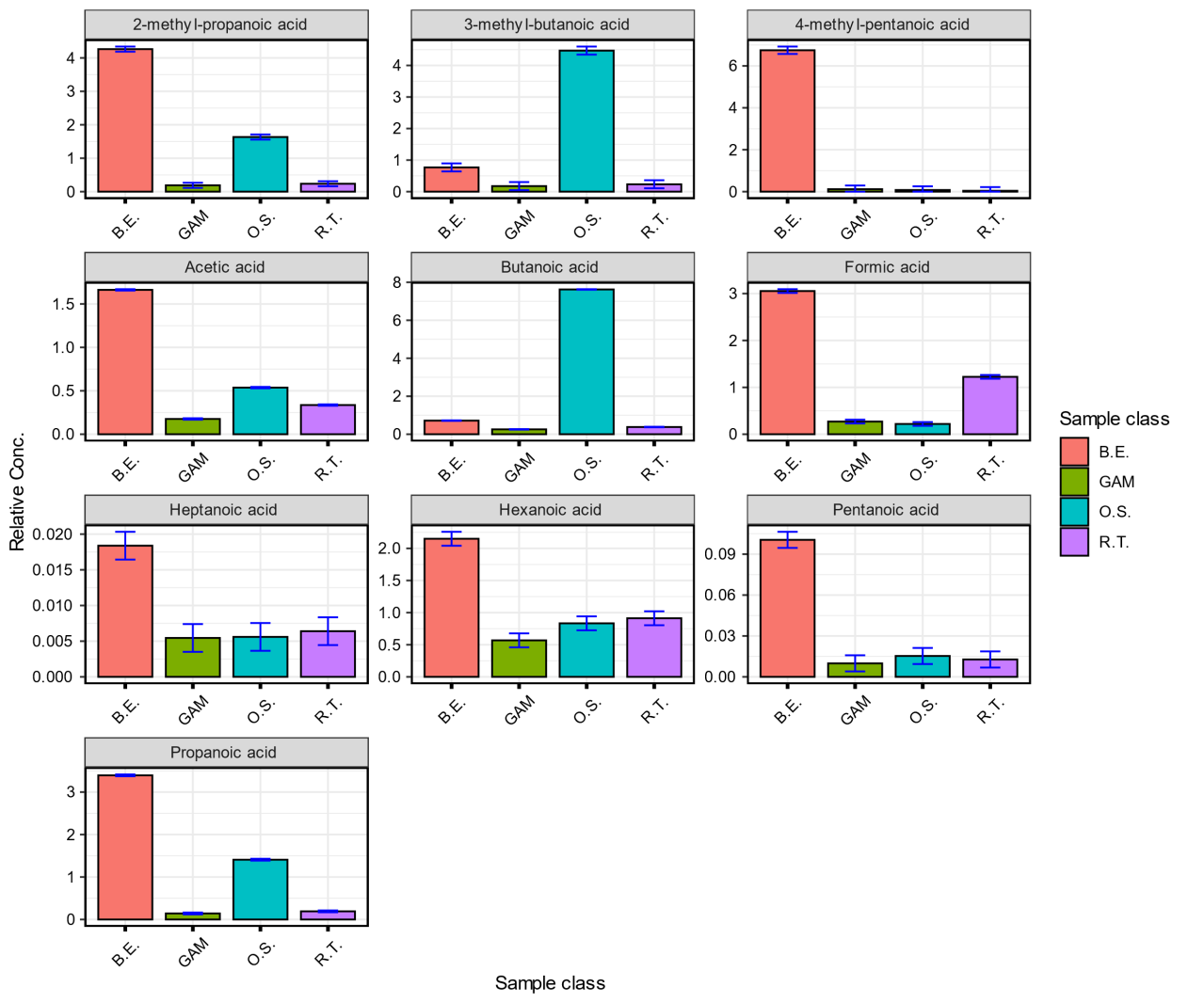


**Suppl. Fig. 23: Relative concentration of short-chain-fatty-acids (SCFA) in bacterial supernatants.** Here, we display all SCFA with significant difference to control samples (fold-change greater 2). Control samples were created by random pooling of sample material (see main manuscript methods for details). We measured the supernatants of *Bacteroides eggerthii* (B.E.), *Odoribacter splanchnicus* (O.S.) and *Ruminococcus [Blautia] torques* (R.T.). OS and BE were the main producer of most of these compounds. OS was by far the strongest producer of butyrate (butanoic acid & 3-methyl butanoic acid). RT produced Formic acid, but not as much BE.


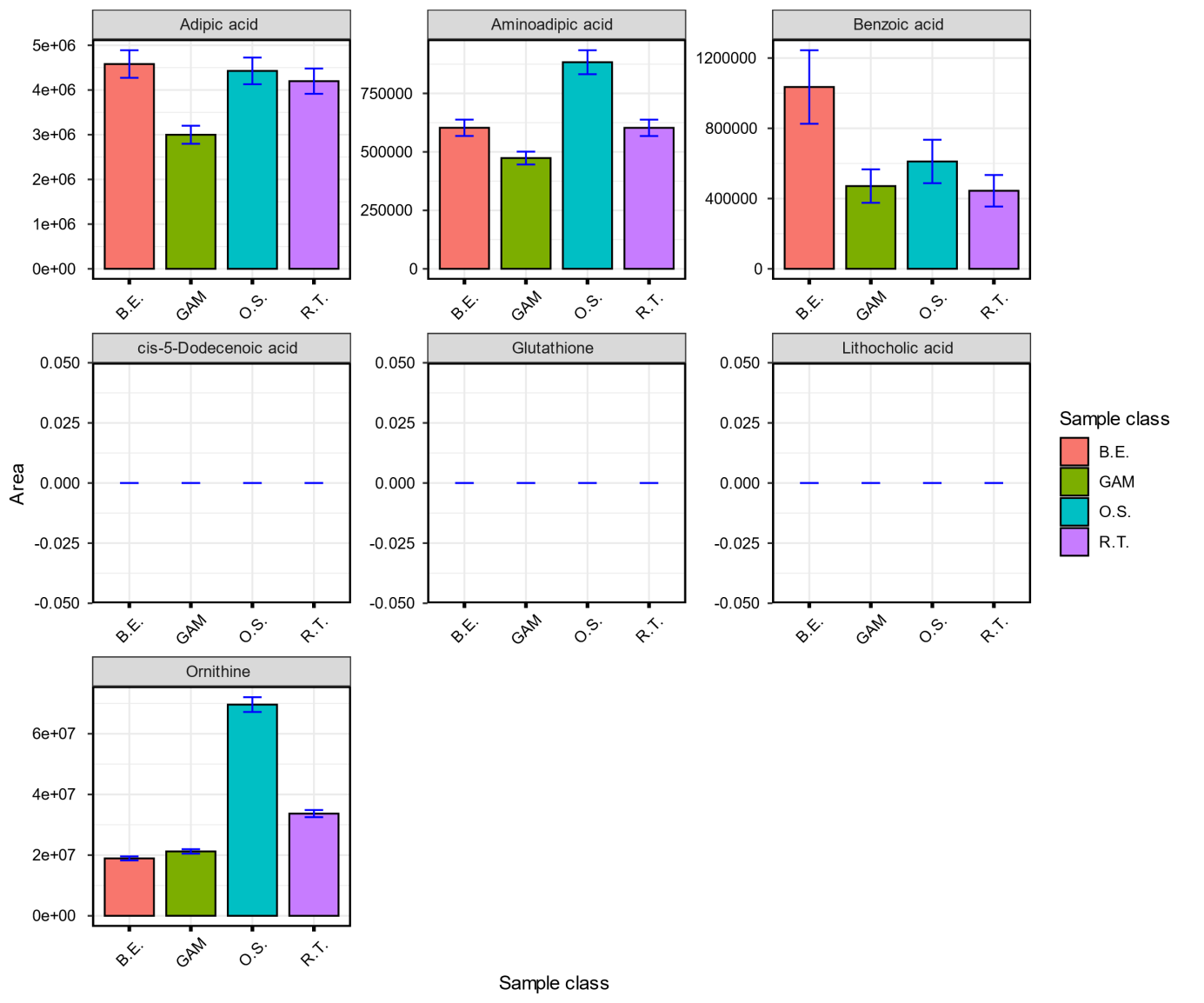


**Suppl. Fig. 24: Area of MS-MS measured metabolites in bacterial supernatants.** Here, we display all non-SCFA metabolites related to the *C. albicans* growth inhibition experiments. Control samples were created by random pooling of sample material (see main manuscript methods for details). We measured the supernatants of *Bacteroides eggerthii* (B.E.), *Odoribacter splanchnicus* (O.S.) and *Ruminococcus [Blautia] torques* (R.T.). Bile acids (Lithocholic acid, cis-5-Dodecenoic acid) were below detection threshold, implying that these species do either (a) not produce such compounds or (b) possible due to missing components in the growth medium used. RT produced Adipic acid is levels comparable to OS and BE. OS produced higher levels of Aminoadipic acid and BE produced higher levels of Benzoic acid. Ornithine was primarily produced by OS, but also a little by RT.


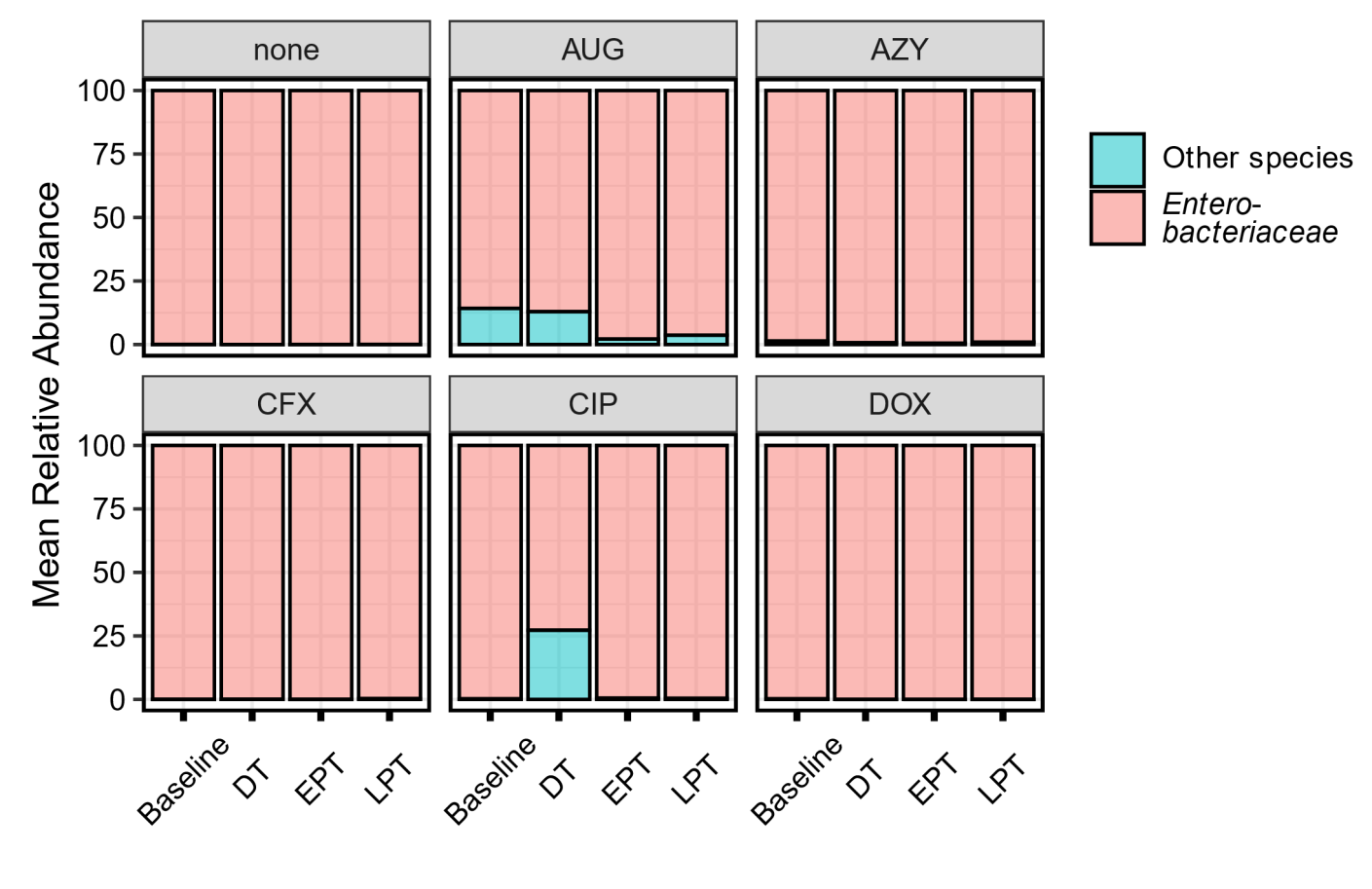


**Suppl. Fig. 25: Mean relative abundance of *Enterobacteriaceae* spp. per ATB.** We investigated the relative abundance of *Enterobacteriaceae* spp. and the impact of antibiotics [ATB] treatment. In most samples, the accumulated relative abundance of *Enterobacteriaceae* spp. (blue) at baseline was below 1%, implying only a minor role for the overall community. The exception were patients treated with AUG, which had roughly 14% *Enterobacteriaceae*. *Enterobacteriaceae* relative abundance in AUG treated patients decreased profoundly at EPT and also LPT compared to baseline levels. In CIP, we observed a strong increase of up to 27% during treatment, but far below 1% for time points before and after treatment.
